# STING induces LUBAC-mediated synthesis of linear ubiquitin chains to stimulate innate immune signaling

**DOI:** 10.1101/2023.10.14.562349

**Published:** 2023-10-16

**Authors:** Tara D. Fischer, Eric N. Bunker, Peng-Peng Zhu, François Le Guerroué, Eunice Dominguez-Martin, Francesco Scavone, Robert Cohen, Tingting Yao, Yan Wang, Achim Werner, Richard J. Youle

**Affiliations:** 1 Biochemistry Section, Surgical Neurology Branch, National Institute of Neurological Disorders and Stroke, National Institutes of Health; Bethesda, MD, USA.; 2 Department of Biochemistry and Molecular Biology, Colorado State University; Fort Collins, CO, USA.; 3 Mass Spectrometry, National Institute of Dental and Craniofacial Research, National Institutes of Health; Bethesda, MD, USA.; 4 Stem Cell Biochemistry Unit, National Institute of Dental and Craniofacial Research, National Institutes of Health; Bethesda, MD, USA.

## Abstract

STING activation by cyclic dinucleotides in mammals induces interferon- and NFκB -related gene expression, and the lipidation of LC3B at Golgi membranes. While mechanisms of the interferon response are well understood, the mechanisms of NFκB activation mediated by STING remain unclear. We report that STING activation induces K63- and M1-linked/linear ubiquitin chain formation at LC3B-associated Golgi membranes. Loss of the LUBAC E3 ubiquitin ligase prevents formation of linear, but not K63-linked ubiquitin chains or STING activation and inhibits STING-induced NFκB and IRF3-mediated signaling in monocytic THP1 cells. The proton channel activity of STING is also important for both K63 and linear ubiquitin chain formation, and NFκB- and interferon-related gene expression. Thus, LUBAC synthesis of linear ubiquitin chains regulates STING-mediated innate immune signaling.

## Introduction

The evolutionarily conserved cGAS-STING pathway initiates potent innate immune responses through several signaling cascades following the detection of double-stranded DNA (dsDNA) in the cytoplasm of cells (1–3). In mammals, cGAS synthesis of the cyclic dinucleotide 2’3’ cyclic GMP-AMP (cGAMP) and its binding to STING at ER membranes induces the trafficking of STING from the ER to the Golgi apparatus. Following trafficking through Golgi compartments, STING initiates a broad transcriptional program of type I interferons mediated by its interaction with the kinase TBK1 and the transcription factor IRF3. At Golgi membranes, active STING also induces the lipidation of the ubiquitin-like protein, LC3B, through mechanisms that are distinct from the known role of LC3B lipidation in autophagosome formation (4–6). Activation of cGAS-STING also induces the transcription of NFκB related genes through poorly understood mechanisms (7–11). Although all of these downstream signaling events mediated by STING activation have been reported to play a role in antiviral defense (4, 7, 11–13), whether and how they are mechanistically related is unclear. Here, we report that ubiquitin robustly co-localizes with activated STING and LC3B at Golgi membranes. As ubiquitylation is important in both autophagy-related and innate immune signaling, we sought to determine whether these ubiquitylation events play a role in STING-mediated innate immune responses.

### cGAMP induces linear and K63 ubiquitin chain formation.

**Figure 1.**
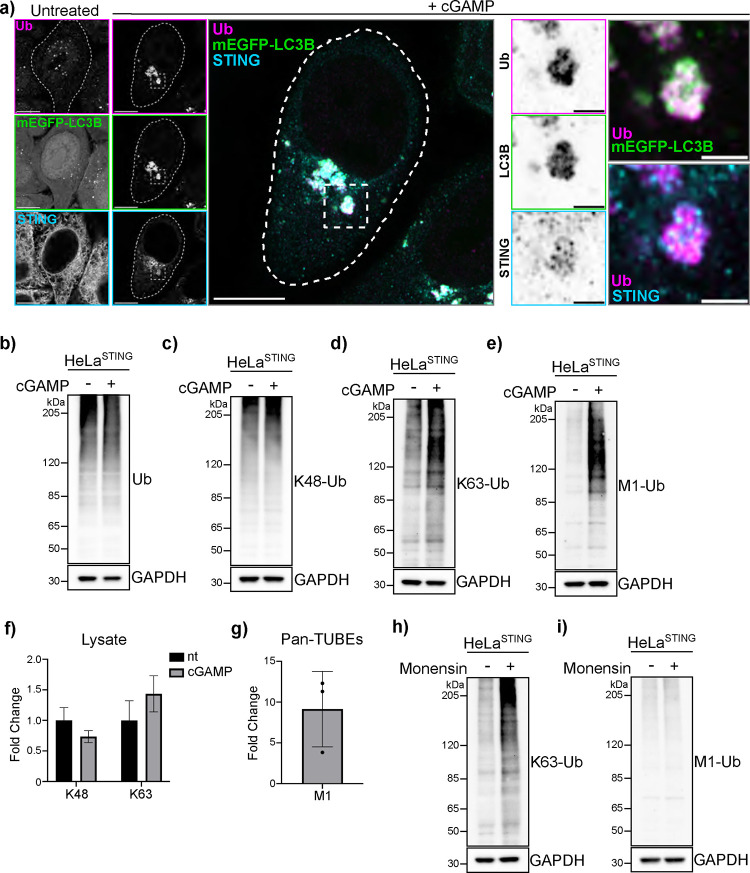
cGAMP induces linear and K63 ubiquitin chain formation. **a)** Representative Airyscan-processed confocal images of Wild-type (WT) HeLa cells stably expressing BFP-P2A-STING (HeLa^STING^) and mEGFP-LC3B (green) treated with 60 μg/mL of cGAMP for 8 hours prior to PFA-fixation and immunostaining with antibodies raised against mono- and poly-ubiquitin chains (Ub; magenta) and STING (cyan). Scale bar = 10 μm, and 2 μm (inset). Imaging was replicated in 3 independent experiments. **b-e)** Representative immunoblots of indicated proteins detected in HeLa^STING^ cell lysates prepared after treatment with 120 μg/mL cGAMP for 8 hours. Immunoblotting was replicated in 3 independent experiments. **f-g)** Quantification of ubiquitin-GG linked peptides from lysate (f) and Pan-TUBE (g; Tandem-Ubiquitin Binding Entities) enriched samples identified by targeted LC-MS/MS. HeLa^STING^ cells stably expressing mEGFP-LC3B were treated with 120 μg/mL of cGAMP for 8 hours prior to cell lysis, Pan-TUBE enrichment, and LC-MS/MS analysis. Mass spectrometry data from cell lysates is from 2 independent experiments with 3–5 technical replicates each. Pan-TUBE enrichment was performed in technical triplicate from one of the same cell lysates analyzed in panel f. **h-i)** Representative immunoblots for indicated linkage specific ubiquitin chains in HeLa^STING^ cell lysates prepared after treatment with 100uM Monensin for 1 hour. Immunoblotting was replicated in 3 independent experiments.

Activation of STING and its trafficking to the perinuclear region of cells induces clusters of small LC3B positive vesicles near the Golgi apparatus (4, 5). Immunostaining for ubiquitin (Ub) eight hours after cGAMP treatment in HeLa cells stably expressing untagged STING (HeLa^STING^) and mEGFP-LC3B shows that >80% of cells present Ub positive foci (Fig. S1a–b) that co-localize with mEGFP-LC3B (Fig. 1a and Fig. S1a–c), and a subset of STING punctae (Fig. 1a) in the perinuclear region.

Ubiquitin can be conjugated to other ubiquitin molecules at any of the seven lysine residues and to the N-terminal methionine (M1), forming unique polyubiquitin chains that mediate specific downstream signaling pathways (14). We used linkage specific ubiquitin antibodies to immunostain for K48- and K63-Ub chains (Fig. S1d–f). Over 80% of mEGFP-LC3B foci stain for K63-Ub (Fig. S1f), while no K48-Ub signal is detected (Fig. S1d and f). K63-Ub co-localization with LC3B can also be detected using a fluorescently tagged probe that selectively binds K63-Ub chains (15) (Movie 1 and Fig. S1g). Live cell imaging shows a correlation between the K63-Ub sensor and mScarletI-LC3B starting around two hours and increasing over time after treatment with cGAMP (Movie 1 and Fig. S1g). Consistently, a cGAMP-induced increase in K63-Ub, but not K48-linked or total-Ub, can be detected by immunoblotting (Fig. 1b–d). We also probed for M1-linked (hereafter also referred to as linear) ubiquitin chain formation following cGAMP treatment using both a commercially available and a custom antibody (16). cGAMP induces a robust increase in M1-Ub chains detected using both antibodies (Fig. 1e and Fig. S1h). Further, we used mass spectrometry to assess cGAMP-induced ubiquitin chain linkages. Detection of GG-K/M linked peptide abundances in cell lysates shows a small increase in GG-K(63) peptides, and no change in GG-K(48) peptides, upon cGAMP treatment (Fig. 1f). Linear ubiquitin chain linkages were detected after enrichment for ubiquitin using Pan-TUBE (Tandem Ubiquitin Binding Entities) pulldown, showing an eight-fold average increase in GG-M(1) peptide abundance with cGAMP treatment compared to the untreated control (Fig. 1g).

LC3B lipidation at acidic organelles can be induced by a variety of stimuli, including lysosomotropic agents, such as Monensin (also referred to as CASM) (17). To determine whether ubiquitin co-localization with LC3B is a general feature of LC3B lipidation at acidic organelles, we immunostained for ubiquitin following Monensin treatment in HeLa^STING^ cells stably expressing mEGFP-LC3B. Ubiquitin is detected at some, but not all, Monensin-induced LC3B vesicles (Fig. S2a). Immunoblotting for K63- and M1-Ub chain formation following Monensin treatment shows an increase in K63-Ub chains, but not M1-Ub chains (Fig. 1h–i), indicating that linear ubiquitin chain formation may be selectively associated with STING-mediated LC3B lipidation at Golgi membranes.

### LUBAC mediates cGAMP induced linear ubiquitin chain formation.

**Figure 2.**
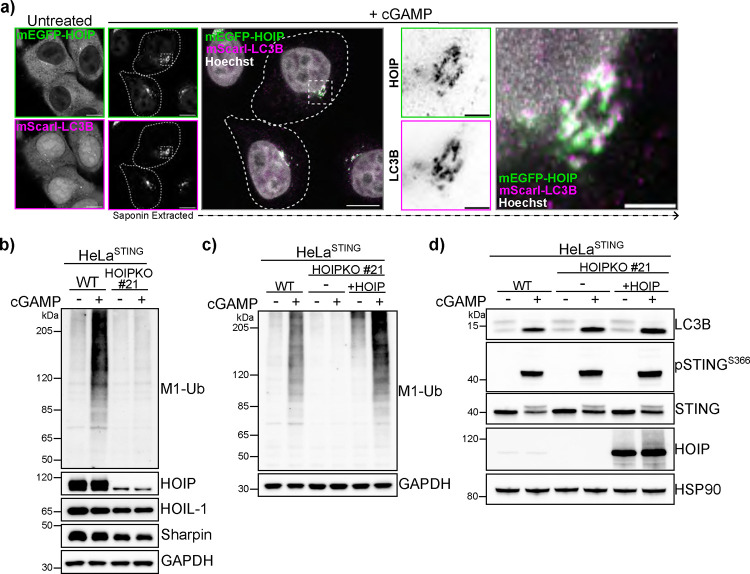
LUBAC mediates cGAMP induced linear ubiquitin chain formation. **a)** Representative Airyscan-processed confocal images of HeLa^STING^ cells stably expressing mScarletI-LC3B (magenta) and mEGFP-HOIP (green) treated with 120 μg/mL of cGAMP for 8 hours prior to saponin extraction and PFA-fixation. Scale bar = 10 μm, and 2 μm (inset). Imaging was replicated in 2 independent experiments. **b-d)** Representative immunoblots of indicated proteins detected in HeLa cell lysates from WT^STING^, HOIPKO^STING^ (Clone #21), and HOIPKO^STING^ (Clone #21) stably expressing untagged HOIP (c & d) prepared following treatment with 120 μg/mL cGAMP for 8 hours. Immunoblotting was replicated in 3 independent experiments.

While many E3 ligases generate K63 ubiquitin chains, linear ubiquitin chains are only known to be formed by the E3 ligase HOIP, a component of the Linear Ubiquitin Assembly Complex (LUBAC) (18, 19). Stable overexpression of mEGFP tagged HOIP in HeLa^STING^ cells also stably expressing mScarletI-LC3B, shows a cytosolic localization in untreated cells, and a clustering of mEGFP-HOIP in the perinuclear region, co-localizing with mScarletI-LC3B foci upon treatment with cGAMP (Fig. 2a). To determine whether HOIP is required for cGAMP induced linear ubiquitin chain formation, we generated a knockout HeLa cell line (HOIPKO HeLa^STING^). cGAMP induced M1-Ub chains are eliminated in HOIPKO HeLa^STING^ cells (Fig. 2b). Stable reconstitution of HOIPKO HeLa^STING^ cells with untagged HOIP rescues M1-Ub chain formation induced by cGAMP treatment (Fig. 2c). Stable overexpression of the linear ubiquitin-specific deubiquitylase OTULIN in HeLa^STING^ cells, which primarily regulates linear ubiquitin dependent LUBAC activity (20, 21), also eliminates M1-Ub chains induced by cGAMP (Fig. S3a). Notably, detection of cGAMP induced K63-Ub chains (Fig. S3b–c), LC3B lipidation (Fig. 2d and Fig. S3d), and phosphorylation of STING at S366 (Fig. 2d and Fig. S3d) are all unaffected by the loss of linear ubiquitin chain formation either by HOIP KO or OTULIN overexpression. Degradation of endogenous STING is also unaffected in HOIPKO cells compared to WT cells (Fig. S3e). Collectively, these results demonstrate that linear ubiquitin chain formation induced by cGAMP activation of STING is mediated by LUBAC, and is not required for K63 ubiquitin chain formation, LC3B lipidation, or STING activation and degradation.

### UBAN-domain proteins, TNIP1 and OPTN, co-localize with cGAMP induced LC3B foci.

**Figure 3.**
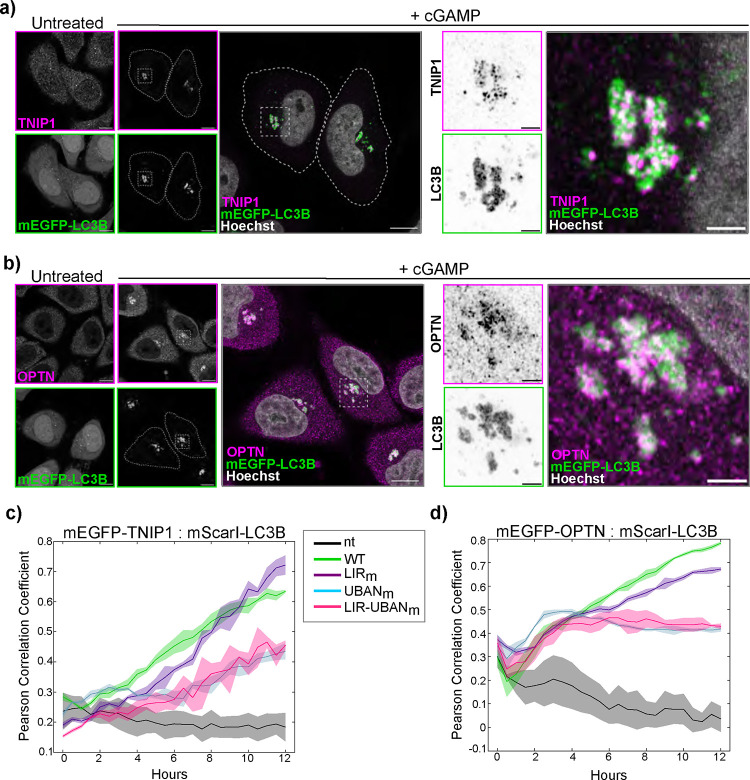
UBAN-domain proteins, TNIP1 and OPTN, co-localize with cGAMP induced LC3B foci. **a-b)** Representative Airyscan-processed confocal images of WT HeLa^STING^ cells stably expressing mEGFP-LC3B (green) treated with 120 μg/mL of cGAMP for 8 hours prior to PFA-fixation and immunostaining with antibodies raised against TNIP1 (a) or OPTN (b) (magenta). Scale bar = 10 μm, and 2 μm (inset). Imaging was replicated in 3 independent experiments. **c-d)** Pearson correlation coefficient of mScarletI-LC3B and mEGFP-TNIP1-WT or mutants (LIR_m_(F83A, L86A, F125A, V128A), UBAN_m_(D472N), and LIR-UBAN_m_ (F83A, L86A, F125A, V128A and D472N)) (**c**) or OPTN-WT or mutants (LIR_m_(F177A and E179A), UBAN_m_(D474N), and LIR-UBAN_m_ (F177A, E179A and D474N)) (**d**) over the time course of treatment in live cell imaging experiments. Cells were treated with 120 ug/mL cGAMP and imaged on a spinning disk confocal microscope for 12 hours. Representative images in Supp. Fig. 4a–b. Quantification is from 3 wells analyzed in the same experiment. Imaging was replicated in 2 independent experiments.

Proteins that bind ubiquitin chains play essential roles in both canonical autophagy and innate immune signaling (22). Some ubiquitin binding proteins also have LC3B/ATG8 interacting motifs (LIRs) that contribute to cargo targeting and autophagosome biogenesis in selective autophagy (23). UBAN (Ubiquitin Binding domain in ABIN1 and NEMO) domains bind linear ubiquitin chains with high affinity, as well as K63 ubiquitin chains (24–26). TNIP1 (also known as ABIN1) and OPTN are UBAN domain-containing proteins that also have LIR motifs and play roles in both selective autophagy and innate immune signaling (27, 24, 28–32). As STING activation induces the spatial co-localization of LC3B and ubiquitin, we asked whether TNIP1 and OPTN are also localized at these sites. Immunostaining for TNIP1 and OPTN in HeLa^STING^ cells stably expressing mEGFP-LC3B shows that endogenous TNIP1 and OPTN are detected at LC3B foci by immunostaining following cGAMP treatment (Fig. 3a–b). To determine whether the UBAN domain or LIR motif contribute to mEGFP-TNIP1 and -OPTN co-localization with LC3B foci, we mutated key residues in these regions individually, and in combination, and examined co-localization with LC3B over time after cGAMP treatment. Live cell imaging of HeLa^STING^ cells stably expressing mScarletI-LC3B and mEGFP tagged TNIP1 or OPTN demonstrate that co-localization of both with LC3B begins to increase at two hours (Fig. 3c–d), similarly to the K63-Ub sensor (Fig. S1g). The co-localization of the TNIP1 UBAN and double mutants with LC3B increases over time of cGAMP treatment, but not as substantially as WT and LIR mutant TNIP1 levels (Fig. 3c, Fig. S4a). The co-localization kinetics of the OPTN UBAN mutants with LC3B decreases relative to WT and LIR mutants following six hours of cGAMP treatment (Fig. 3d, Fig. S4b). These data indicate that the ubiquitin binding through the UBAN domain may be involved in TNIP1 and OPTN co-localization with LC3B foci. Linear ubiquitin chain formation, however, is not required for TNIP1- and OPTN- co-localization with cGAMP-induced LC3B foci as both colocalize with mEGFP-LC3B similarly in HOIPKO HeLa^STING^ cells compared to WT (Fig. S4c–e).

### Linear ubiquitin chains play a role in STING-mediated NFκB- and interferon-related immune signaling.

**Figure 4.**
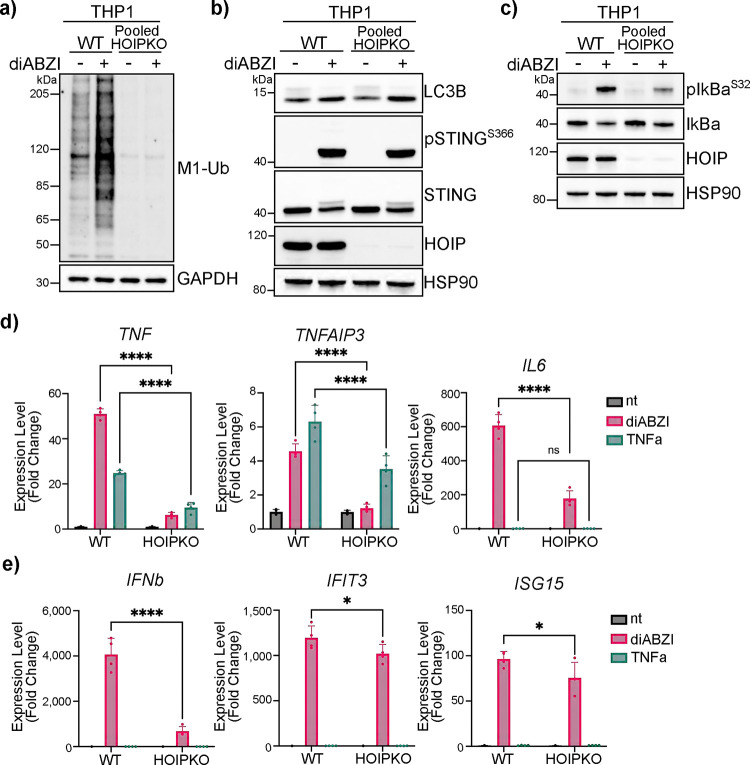
Linear ubiquitin chains play a role in STING-mediated NFκB- and interferon-related immune signaling. **a-c**) Representative immunoblots of indicated proteins detected in lysates from WT and pooled HOIP KO THP1 cells prepared following treatment with 1μM diABZI for 4 hours. Immunoblotting was replicated in 3 independent experiments. **d-e**) Relative expression changes of indicated NFκB- (d) and interferon-related (e) genes detected by quantitative RT-PCR in THP1 cells treated with 10ng/mL TNFα for 30 minutes or 1μM diABZI for 4 hours. Quantification of relative expression is from 4 independent experiments analyzed at the same time. A 2-way ANOVA with a Tukey’s multiple comparisons test was performed on 2^−ΔΔCt^ values. Error bars represent Standard Deviation. *<0.05, **<0.01, ***<0.001, ****<0.0001.

Whereas K63 ubiquitin chains have been associated with diverse cellular functions, linear ubiquitin chain formation by LUBAC is most widely understood to function in immune signaling, particularly through the NFκB pathway (33, 26, 34, 19). Since activation of STING induces NFκB signaling, in addition to Type I IFN signaling, we asked whether linear ubiquitin chain formation induced by cGAMP treatment mediates NFκB signaling in both THP1 monocytes and HeLa cells. In THP1 cells, treatment with the membrane permeable STING agonist, diABZI, induces the HOIP-dependent formation of M1-Ub chains (Fig. 4a). diABZI-induced phosphorylation and degradation of endogenous STING, and LC3B lipidation is not different in HOIPKO THP1 compared to WT (Fig. 4b); thus, the activation of STING is not affected by HOIPKO in THP1 cells, consistently with HOIPKO in HeLa cells (Fig. 2d and Fig. S3e). However, loss of HOIP decreases diABZI-induced phosphorylation of the NFκB inhibitor, IκBα (Fig. 4c), a modification required for NFκB-related transcriptional regulation (35). Consistent with a decrease in activation of the NFκB pathway, diABZI-induced transcription of the NFκB-related genes, *TNF* and *TNFAIP3*, is also reduced in HOIPKO THP1 cells, similarly to TNFα-induced NFκB gene transcription (Fig. 4d). Interestingly, the diABZI-induced expression of the IRF3-mediated gene *IFNβ* and downstream interferon-stimulated genes, *IFIT3* and *ISG15*, are also reduced in HOIPKO THP1 cells (Fig. 4e). This may reflect a potential role of NFκB signaling participating to some extent in IRF3-mediated gene induction, in line with previous reports (8). As in THP1 cells, TNFα induced transcription of NFκB related genes is inhibited in HOIPKO HeLa cells (Fig. S5a). However, HOIP and linear ubiquitin chain formation in HeLa cells are not required for the phosphorylation of IκBα (Fig. S5b), or the transcription of NFκB-related (Fig. S5c), and IRF3- and interferon-related genes (Fig. S5d). A modest decrease in *TNF, IFNb, and IFIT3* gene expression, but not others, is found in HeLa cells depleted in linear ubiquitin chains by the overexpression of OTULIN (Fig. S6). These data indicate that HOIP-mediated linear ubiquitin chain formation is important for STING activation induced NFκB and interferon-related signaling in THP1 monocytes, however there may be distinct mechanisms in HeLa cells.

### The channel activity of STING is important for linear and K63 ubiquitin chain formation and innate immune signaling.

**Figure 5.**
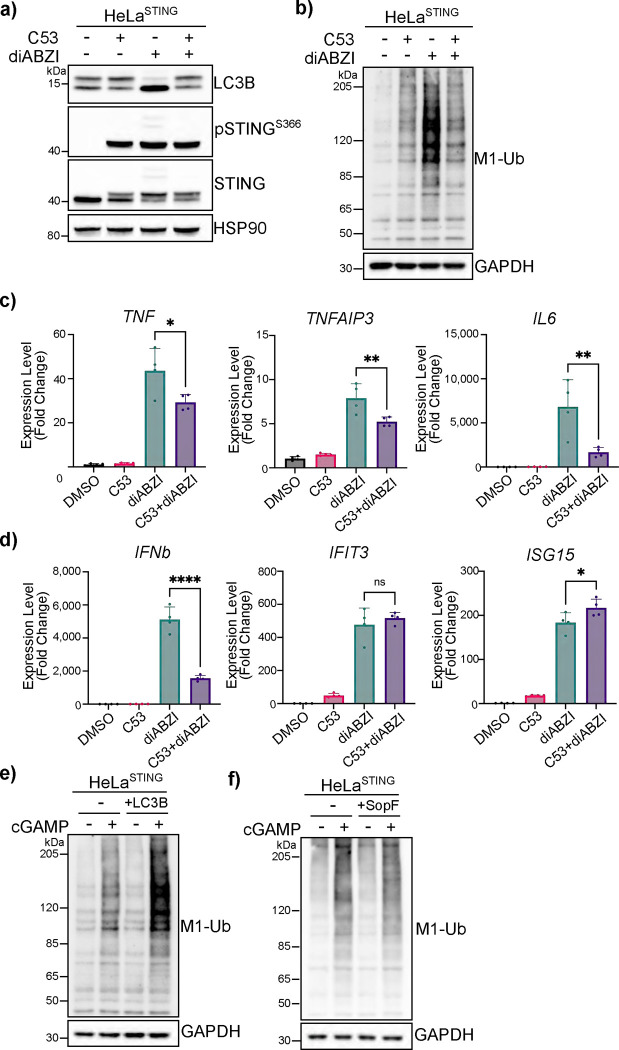
The channel activity of STING is important for linear and K63 ubiquitin chain formation and innate immune signaling. **a-b)** Representative immunoblots of indicated proteins detected in HeLa^STING^ cell lysates prepared after treatment with either DMSO, 10uM C53, 1uM diABZI, or both C53 and diABZI for 4 hours. Immunoblotting was replicated in 3 independent experiments. **c-d**) Relative expression changes of indicated NFκB-related genes (c) and interferon-related genes (d) detected by quantitative RT-PCR in THP1 cells treated with DMSO, 10uM C53, 1uM diABZI, or both C53 and diABZI for 4 hours. Quantification of relative expression is from 4 independent experiments analyzed at the same time. A one-way ANOVA with a Tukey’s multiple comparisons test was performed on 2^−ΔΔCt^ values. Error bars represent Standard Deviation. *<0.05, **<0.01, ***<0.001, ****<0.0001. **e-f**) Representative immunoblots of indicated proteins detected lysates from HeLa^STING^ and HeLa^STING^ cells with stable overexpression of mEGFP-LC3B (e) or mEGFP-SopF (f) prepared after treatment with 120 μg/mL cGAMP for 8 hours. Immunoblotting was replicated in 3 independent experiments.

It was recently reported that STING forms a proton channel that disrupts the lumenal pH of the Golgi following activation and trafficking (36). This channel activity is required for STING-mediated LC3B lipidation at Golgi membranes, NLRP3-related immune signaling, and cell death (37), but not for the phosphorylation of STING by TBK1 or its trafficking. The delineation of downstream signaling events mediated by STING was demonstrated by use of the agonist, C53, that binds the ER-lumenal facing transmembrane (TM) region of STING and is thought to promote its oligomerization and trafficking (38). However, the binding of C53 to the TM region blocks the channel that forms in the membrane (36). Therefore, we asked whether the channel inhibitor, C53, affects ubiquitin chain formation. In HeLa^STING^ cells, C53 alone induces the trafficking (Fig. S7a) and phosphorylation of STING, but not LC3B lipidation (Fig. 5a). Combining C53 treatment with diABZI blocks the diABZI-induced LC3B lipidation, but not STING trafficking (Fig. S7a) or phosphorylation (Fig. 5a), as recently reported (36). Strikingly, C53 also blocks diABZI-induced M1-Ub chain formation (Figure 5b). C53 alone moderately promotes M1-Ub chain formation (Figure 5b), indicating that there may be channel-independent linear ubiquitin chain formation induced by STING activation. C53 also completely blocks perinuclear foci formation of the K63-Ub sensor induced by diABZI (Fig. S7b–c). Similarly, diABZI-induced foci formation of endogenous TNIP1 and OPTN is also blocked by C53 (Fig. S8a–d), however some small TNIP1 punctae can still be observed (Fig. S8a). In THP1 cells, C53 alone does not appear to activate STING (Fig. S8e), unlike in HeLa cells (Fig. 5a). The combined treatment of C53 and diABZI, however does block diABZI-induced LC3B lipidation, whereas the S366 phosphorylation of STING is unaffected (Fig. S8e). Although C53 does not appear to affect the diABZI-induced phosphorylation of IκBα (Fig. S8f), it reduces NFκB- and IRF3-related gene expression in THP1 cells (Fig. 5c–d). Therefore, STING channel activity is important for STING-mediated NFκB- and interferon-related innate immune signaling and correlates with both HOIP-mediated linear ubiquitin chain formation and LC3B lipidation. As HOIPKO does not affect LC3B lipidation, we hypothesized that STING-mediated LC3B lipidation may function upstream of the linear ubiquitination and NFκB signaling. Stable overexpression of mEGFP-LC3B in HeLa^STING^ cells increases the detection of M1-Ub following cGAMP treatment (Fig. 5e) and condenses both ubiquitin and STING into foci (Fig. S9a). Stable expression of SopF, a bacterial effector that blocks the recruitment of ATG16L1 to the V-ATPase and LC3B lipidation (5, 39, 40), does not affect M1-Ub chain formation (Fig. 5f), but blocks ubiquitin foci formation following cGAMP treatment (Fig. S9b). Thus, the stimulation of linear ubiquitin chain formation by the overexpression of LC3B, and the inhibition of ubiquitin foci formation by C53 or SopF, that also block LC3B lipidation, suggests that STING activation induced LC3B lipidation may promote ubiquitin chain formation.

## Discussion

Innate immune responses to cytosolic DNA, from viruses, bacteria, mitochondria or the nucleus are mediated in part by cGAS activation of STING (41). Following the detection of dsDNA, mammalian cGAS synthesizes the cyclic dinucleotide, 2’3’ cGAMP, that binds to STING and induces its membrane trafficking from the ER to the Golgi apparatus. STING localization at the Golgi and Golgi-related vesicles, initiates interferon-related immune signaling and LC3B lipidation at Golgi membranes. STING activation also induces NFκB transcriptional responses, which confers an important interferon-independent antiviral signature of STING signaling (7–11). However, the mechanism of NFκB activation by STING, and the relation to other signaling cascades induced by STING activation and trafficking are unclear. We find that STING activation induces the localization of the Linear Ubiquitin Chain Assembly Complex (LUBAC) E3 ligase, HOIP, at LC3B-associated Golgi membranes and its synthesis of linear ubiquitin chains to stimulate immune signaling. Loss of HOIP prevents linear ubiquitin chain formation, which impairs STING activation-induced IκBα phosphorylation, and the expression of NFκB-related genes. Interestingly, interferon-related gene expression is also impaired with the loss of HOIP, even though STING phosphorylation by the kinase TBK1 is intact. While a relationship between interferon and NFκB signaling following STING activation has been long appreciated (8), it will be important to further understand the mechanistic role of LUBAC and linear ubiquitin chains in interferon responses.

K63 ubiquitin chains are also localized with LC3B at the Golgi upon STING activation, which raises several intriguing questions regarding whether these ubiquitin chains are conjugated to specific substrates, whether they are homotypic or heterotypic (i.e., hybrid K63-M1 chains), and whether they play specific or redundant roles in STING-induced innate immune signaling. Indeed, STING itself is reported to be conjugated to K63 ubiquitin chains (42–44). It will be important to unravel the specific characteristics of these ubiquitin chains and how they regulate the cGAS-STING pathway. Further, it will be exciting to determine whether K63 and linear ubiquitin chains mediate differential recruitment of ubiquitin binding proteins that regulate immune signaling similarly to other pathways (45, 46). Here we have identified two ubiquitin binding proteins, TNIP1 and OPTN, that co-localize at LC3B foci with ubiquitin after STING activation. TNIP1, and its interacting partner, the deubiquitylase A20, are known negative regulators of NFκB signaling (27, 24, 28, 29), and thus may be involved in STING-mediated immune responses. OPTN constitutively interacts with TBK1, an important feature of the function of OPTN and TBK1 in other pathways such as mitophagy (47). TBK1 recruitment to STING at Golgi membranes may be mediated by both STING and OPTN to regulate downstream immune signaling.

In addition to revealing a key mechanism for STING-mediated innate immune signaling, our work also extends the LUBAC activity to an intracellular Golgi-derived vesicle platform. The role of LC3B lipidation at Golgi vesicles in proximity to ubiquitin chains is intriguing. The STING-mediated LC3B lipidation at Golgi membranes, we called VAIL (V-ATPase induced LC3B lipidation) (5), resembles single membrane lipidation of ATG8s called CASM (Conjugation of ATG8s at Single Membranes) or noncanonical autophagy, known to be induced by pharmacological and protein-mediated ionic flux (48). This similarity includes the dependence on the V-ATPase, the C-terminal WD40 domain of ATG16L1, and the lipidation of ATG8s at acidic organelles (49, 5, 50). Indeed, our previous work (5) suggested that STING may perturb ionic balances in the Golgi following activation, which has recently been shown directly (36). Here we show that, unlike ubiquitin chain formation associated with LC3B lipidation induced by STING activation, CASM induced by the ionophore, Monensin, induces K63, but not linear ubiquitin chain formation. These results reveal a new distinction between ionophore-induced CASM and STING activation-induced LC3B lipidation. Further, we show that inhibition of the recently reported channel activity of STING by C53, also inhibits STING-mediated linear and K63 ubiquitin chain formation, and NFκB- and interferon-related gene expression. The correlations between 1) C53 inhibition of LC3B lipidation and ubiquitin chain formation, 2) the amplification of linear ubiquitin chain formation and condensation of ubiquitin and STING in perinuclear foci with overexpression of LC3B, and 3) the spatial co-localization of LC3B, ubiquitin, and HOIP in discrete foci, support a new model for LC3B lipidation at Golgi membranes playing a role in the local formation of ubiquitin chains following STING activation. As both the V-ATPase-ATG16L1 axis of LC3B lipidation (39) and LUBAC-related xenophagy- and NFκB-signaling restricts *Salmonella* proliferation (51), it will be interesting to determine the role of STING-mediated LC3B and ubiquitin chains at Golgi membranes in cell autonomous immunity. In conclusion, our work has revealed several new mechanistic insights into how STING activates innate immune signaling, and the potential relationship between the multiple signaling cascades involved in antiviral defense.

## Materials and Methods

### Reagents and Resources

Please see complete list of materials with experimental details in supplemental file 1.

### Cell Culture

HeLa (ATCC) and HEK293T (ATCC) cells were cultured in Dulbecco’s Modified Eagle Medium (DMEM; Thermofisher) supplemented with 10% (v/v) Fetal Bovine Serum (FBS; R&D), 1 mM Sodium Pyruvate (Thermofisher), and 2 mM GlutaMAX (Thermofisher). THP1 (Synthego-ATCC) cells were cultured in Roswell Park Memorial Institute (RPMI) 1640 (ATCC). HeLa FRT/TREX cells stably expressing an inducible version of the Vx3-EGFP sensor previously reported in (15) with a DD degron fused to the N-terminus (pcDNA5-FRT/TO-DD-Vx3-EGFP) were kindly provided by Tingting Yao and Robert Cohen.

### Generation of CRISPR Knockout Cell Lines

For HOIPKO HeLa cells, gRNAs were annealed and ligated into a BbsI-digested pSpCas9(BB)-2A-Puro (PX459) V2.0 vector (Addgene #62988). HeLa cells were transfected with the gRNA-containing plasmid using the JetOptimus (Polyplus) transfection reagent, and subsequently treated with 1 ug/ml puromycin (Invivogen) for 2 days to select for cells expressing the plasmid. Isolation of single cells from the puromycin-resistant pool was obtained by limiting dilution and clonal expansion in 96 well plates. Individual clones were then screened for knockout edits by PCR amplification of the target locus from extracted genomic DNA and sanger sequencing of the amplicon. Sequence data was analyzed using the Inference of CRISPR Edits (ICE) tool from Synthego to identify clones with frame-shifting insertions or deletions (InDels). Selected clones were finally validated by measuring protein abundance detected by immunoblotting. HOIPKO THP1 and Mock Transfected WT cells were purchased as an express pooled cell line from Synthego. Details for gRNA sequences for each KO cell line are in Supplemental File 1.

### Cloning and Generation of Stable Cell Lines

pHAGEb vectors were modified from the pHAGE (HIV-1 Gustavo George Enhanced) vector. Plasmids were generated by PCR amplification of open reading frames (ORFs) in cDNA and ligation into a linearized pHAGEb vector using a Gibson assembly kit (New England Biolabs). dH5a or Stable competent E. coli cells (New England Biolabs) were then transformed with assembled plasmids and grown on LB agar plates with appropriate antibiotics. Single colonies were selected, expanded in antibiotic containing liquid culture, and screened for successful insertion of the amplicon by diagnostic restriction enzyme digest. Selected plasmids were further validated by sanger sequencing. Complete plasmid sequences are available upon request.

HeLa cell lines with stable overexpression of indicated genes were generated by lentivirus infection. Lentivirus was generated using HEK293T cells and lentiviral system plasmids (pHDM-G, pHDM-HGPM2, pHDM-tat1B, and pRC-CMV-rev1B) using a protocol detailed in Wang, 2020 (52). HEK293T cells were transfected with the lentiviral system plasmids (500ng each), plasmid DNA (1ug) and PEI prepared in OptiMEM media (Gibco). Following an initial exchange for fresh media 16–24 hours after transfection, lentiviral conditioned media was harvested 28–72 hours post-transfection, and filtered through a 0.45um PVDF syringe filter prior to transduction of target cells. Target cells were transduced by incubating cells with generated lentivirus and 8 ug/mL Polybrene for 24 hours. At least three days following transduction, generated stable cell lines were sorted based on the expressed fluorescent proteins using Fluorescence-Activated Cell Sorting (FACS) to obtain homogenous cell populations at empirically determined optimal protein expression.

### Immunofluorescence

Cells were plated onto #1.5 chambered coverglass (Cellvis) or in 96 well glass plates (DOT Scientific Inc) 18–24 hours before experiments were performed. Following indicated treatments, cells were fixed with prewarmed 4% paraformaldehyde (PFA; Electron Microscopy Services) for 10–15 minutes at 37°C. For general immunofluorescence procedures, fixed cells were rinsed with 1xPBS and permeabilized with 0.5% Triton-X 100 (Sigma) in 1xPBS for 5 minutes prior to blocking for 1 hour in buffer containing 3% goat serum, 1% BSA, and 0.1% Tween-20 in 1xPBS. Cells were then incubated with primary antibody prepared in blocking buffer overnight at 4°C and then washed with 1xPBS prior to incubation with secondary antibodies in blocking buffer for 1 hour at room temperature.

For linkage-specific ubiquitin antibodies, cells were incubated in blocking buffer overnight at 4°C, and then incubated in primary antibodies prepared in blocking buffer for 1 hour at room temperature prior to the washing and secondary antibody incubation steps per the recommended protocol described in Newton et al., 2012 (53). Following immunostaining, cells were stained with Hoechst dye prior to imaging.

For saponin extraction, cells were rinsed with ice cold HBSS, permeabilized with Saponin Extraction Buffer (SEB; 80mM PIPES, pH 6.8, 1mM MgCl_2_, 1mM EGTA, 0.1% Saponin (w/v)) for 2 minutes, then 4 minutes on ice, and washed again 2x with HBSS prior to fixation as described above (46).

### Live Cell Imaging

For live cell imaging, cells were plated in 96 well glass plates (DOT Scientific Inc) 18–24 hours before experiments were performed. For Vx3-EGFP (K63-Ub Sensor) cells were treated with Doxycycline (1 ug/mL) and the Shield1 ligand (500nM) at the time of plating and at least 24 hours prior to experiments. Image acquisition began immediately after indicated treatments and occurred every 30 minutes over a 12-hour time course. Imaging was performed in a live-cell chamber at 37°C, 5% CO_2_, and constant humidity.

### Microscopy

For super-resolution imaging, fixed and/or immunostained cells were imaged on a Zeiss LSM 880 Airyscan Confocal Microscope using a Zeiss 63× 1.4 NA Plan-Apochromat objective, equipped with a Piezo high-precision stage at room temperature. Z-stack images were acquired with 405-, 488-, 561-, and 633-nm lasers, MBS 488/561/633 and MBS 405 beam splitters, Zeiss BP 570–620 + LP 645, BP 420–480 + BP 495–550, and BP 420–480 + LP 605 emission filters, and the Airyscan detector in frame scan mode using the Zen software platform (Carl Zeiss Microscopy). Images were then processed for Airyscan 3D deconvolution using Zen software and prepared for publication using FIJI open-source software (54). Upper limits of the pixel display range were adjusted to improve brightness in representative images.

For quantitative and live cell imaging, fixed and immunostained cells, or live cells were imaged on a Nikon Ti-2 CSU-W1 spinning disk confocal microscope using a 40× 1.15 NA water objective, equipped with an automated TI2-N-WID water immersion dispenser. Images were acquired with 405-, 488-, 561-, and 633-nm lasers and 698/70 nm emission filter using the Nikon elements AR microscope imaging software.

### Image Analysis

For Pearson Correlation Coefficient analysis, custom MATLAB scripts were used that performed automated background subtraction and cell segmentation by intensity and size. Individual cells were segmented by performing a gaussian blur on the LC3B channel, then performing a watershedding algorithm to separate the blurred peaks. The separating lines from watershedding were then imposed on the cell mask to separate connected components to isolate single cells. The Pearson’s Correlation Coefficient was calculated for the pixels within each cell, then the median value found for each image. The median value was calculated between each image taken for each well of a 96 well plate, and then averages and standard deviations calculated between wells.

For percent cells with foci, custom MATLAB scripts performed similar processing and cell segmentation to the Pearson’s Correlation analysis, but a secondary segmentation for punctate structures was performed. To find punctae and foci, cell-sized regions were equalized by dividing the image by a gaussian blur of the image, then pixels with high intensity in both the original image and the equalized image found and filtered by size and, in some cases, circularity. The number of pixels positive in the foci mask were then counted for each connected component in the segmented single cell mask and, if above a threshold, were considered cells positive for foci.

For percent foci positive for ubiquitin, we used custom MATLAB scripts to identify LC3B foci as above, then treated each as individual connected components in a loop. Each component underwent a morphological dilation and the original mask subtracted to generate a ‘donut’ shape with no overlapping foci. Then a ratio was found between the foci component and the corresponding dilation to find if there is a higher intensity in the foci compared to near the foci. Foci with a ratio greater than the threshold 1.75 were considered positive for ubiquitin or a specific ubiquitin chain for which they were stained. All MATLAB scripts are available upon request.

### Immunoblotting

Cells were plated in 12-well or 6-well plates 18–24 hours prior to experiments. Following indicated treatments, cells were washed with ice cold HBSS, lysed in ice cold 1x LDS sample buffer (Genscript) supplemented with cOmplete Protease Inhibitor cocktail (Roche), and boiled immediately at 99°C for 10 minutes. Protein quantitation per sample was obtained using Pierce BCA protein assay kit (ThermoFisher). Dithiothreitol (DTT; Sigma) was added to each sample at a final concentration of 100 mM just before gel loading.

For general immunoblotting procedures, 20–30ug of total protein per sample was loaded into wells of 4–12% Bis-Tris gels (Genscript or Invitrogen) and separated in MES or MOPS buffer ran at 65v for 30 minutes, then 125v until the dye front reached the bottom of the gel. Separated proteins were then transferred onto 0.45 um Nitrocellulose membranes in BioRad Transblot Turbo Transfer Buffer with 20% EtOH using the Biorad Trans-Blot Turbo Transfer system (semi-dry). Membranes were blocked in 5% milk 1xPBST at room temperature (RT) for 1 hour prior to primary antibody incubation in 3% BSA 1xPBST at 4°C overnight. Membranes were then thoroughly washed in PBST, incubated in HRP conjugated secondary antibodies raised against the appropriate species in 5% milk PBST for 1 hour at room temperature, and finally washed. HRP signal was developed using either Amersham ECL Prime (Cytiva) or SuperSignal West Femto ECL (Thermo Scientific) and detected using a ChemiDoc Imaging System (BioRad). Images were analyzed using ImageLab (BioRad).

For detection of linkage-specific ubiquitin, a modified version of the protocol described in Newton et al., 2012 (53) was used. 10–15ug total protein was loaded into wells of 3–8% Tris-Acetate gels and separated in tris acetate buffer (Invitrogen) ran at 65v for 30 minutes and then 125v until the dye front reached the bottom of the gel. Separated proteins were then transferred onto 0.45um a Nitrocellulose membrane at 30v for 2 hours in Tris-Glycine buffer (Towbin formulation) supplemented with 10% MeOH using the XCell SureLock blot module system (semi-wet). Membranes were blocked in 5% milk 1xPBST overnight at 4°C, then incubated in primary antibodies in 5% milk 1xPBST for 1 hour at room temperature. Membranes were then thoroughly washed in PBST, incubated in HRP conjugated secondary antibodies raised against the appropriate species in 5% milk PBST for 1 hour at room temperature, and finally washed. HRP signal was developed using SuperSignal West Femto ECL and detected and analyzed as described above.

### LC-MS/MS Quantification of Ubiquitin Linkages

HeLa cells were plated in a 15cm dish ~48 hours prior to experiments. Following treatment, cells were washed 2x with ice cold 1xPBS supplemented with N-Ethymaleimide (NEM; 5mM), scraped from the dish, and centrifuged at 2,000xg for 2 minutes at 4°C. Supernatant was removed, and the cell pellets were resuspended in ice cold lysis buffer (50mM Tris-HCl, pH 7.5, 150mM NaCl, 1% NP-40, 1mM EDTA, 10% Glycerol) supplemented with the deubiquitylase and protease inhibitors (100uM PR-619, 5mM O-Phelanthroline, 5mM NEM, and cOmplete protease inhibitor cocktail). For preparation of peptides from cell lysates, a modified protocol for S-Trap spin column digestion from Protifi was used. ~40ug of protein from the cell lysates was solubilized in either a mixture of 5% SDS, 8M Urea, and 100mM TEAB or 10% SDS and 100mM TEAB, and mixed at 50oC for 5 minutes. After solubilization, samples were reduced with 0.1M TCEP for 15 minutes at room temperature, then alkylated with 0.22M NEM for 15 minutes at room temperature in the dark. Samples were then acidified with 12–21% aqueous phosphoric acid prior to digestion with Trypsin/Lys-C (Promega) and then added immediately to an S-Trap column (Protifi) loaded with S-Trap binding buffer (90% MeOH and 100mM TEAB). Following an initial centrifugation at 4,000×g to trap the proteins, the column was washed with S-Trap Binding Buffer, and then either incubated overnight at 37°C or for 2 hours at 47oC in digestion buffer (Trypsin-LysC and 50mM TEAB) for complete protein digestion. After digestion, the column was rehydrated with 50mM TEAB, and digested peptides were eluted with 0.2% aqueous formic acid, and then a mixture of 0.2% aqueous formic acid and 50% acetonitrile. Peptides eluted from the S-Trap were dried under vacuum and stored at −20oC until analysis. For analysis, each sample was resuspended in 0.1% TFA, a nanodrop reading was taken at UV280 to normalize loading. NanoLC-MS/MS analysis of tryptic peptides was carried out with a Thermo Scientific Fusion Lumos tribrid mass spectrometer interfaced to a UltiMate3000 RSLCnano HPLC system (Thermo Scientific). For each analysis, ~1 μg of the tryptic digest was loaded and desalted in an Acclaim PepMap 100 trapping column (75 μm, 2 cm) at 4 μL/min for 5 min. Peptides were then eluted into an Thermo Scientific Accalaim PepMap^™^ 100 column, (3 μm, 100 Å, 75 μm × 250 mm) and chromatographically separated using a binary solvent system consisting of A: 0.1% formic acid and B: 0.1% formic acid and 80% acetonitrile at a flow rate of 300 nL/min. A gradient was run from 5% B to 37.5%B over 60 minutes, followed by a 5-minute wash step with 90% B and 10-minute equilibration at 5% B before the next sample was injected. The orbitrap Lumos mass spectrometer operated in unscheduled Parallel Reaction Monitor (PRM) mode. Precursor masses were detected in the Orbitrap at R=120,000 (m/z 200). Charge state with the strongest signal of each ubiquitylated ubiquitin peptide was added to the target list. Isolation window was 1.2 m/z. MS/MS spectra were acquired in the orbitrap with R= 30,000 (m/z 200), with AGC target 500%. Collision energy was 28%. Data was processed using Skyline (55) for quantification. Peak detection and integration were manually validated for each peptide before quantification results were exported to Excel.

### Quantitative Real-Time PCR

Total RNA from 5–10×10^5^ cells was extracted using Quick-RNA MiniPrep Plus kit (Zymo Research) followed by reverse transcription using High-Capacity cDNA Reverse Transcription Kit (Thermo Fisher Scientific). Equal amounts of cDNA and corresponding primers were used for qPCR using SYBR Green Master Mix (Thermo Fisher Scientific) and a CFX384 real-time system/C100 Touch Thermal Cycler (Bio-Rad). For each biological sample, the Ct value of the gene interested was normalized against the b-actin Ct to calculate ΔCt. Each ΔCt was normalized to the average ΔCt of untreated samples to generate the ΔΔCt value. Relative gene expression was then analyzed using the 2^−ΔΔCt^ formula and plotted in figures (56). Details for PCR primers can be found in Supplemental File 1.

### Statistics

For quantitative RT-PCR, one way or 2-way ANOVA was performed on the 2^−ΔΔCt^ values with a Tukey’s multiple comparisons test. Statistical analyses were performed using Prism (GraphPad).

## Supplementary Material

Supplement 1

Supplement 2

Supplement 3

## Data Availability

All data are available in the main text or supplementary materials. All cell lines and plasmids are available upon request and materials transfer agreement.
