# Supplementary material for: STING induces HOIP-mediated synthesis of M1 ubiquitin chains to stimulate NFκB signaling
Source: bioRxiv. 2024 Oct 1:2023.10.14.562349. Preprint. [Version 3] doi: 10.1101/2023.10.14.562349 (PMC10592814; doi:10.1101/2023.10.14.562349)
Supplement: Supplement 2 [file NIHPP2023.10.14.562349v3-supplement-2.pdf]

bioRxiv preprint doi: https://doi.org/10.1101/2023.10.14.562349; this version posted October 1, 2024. The copyright holder for this preprint (which was not certified by peer review) is the author/funder. This article is a US Government work. It is not subject to copyright under 17 USC 105 and is also made available for use under a CC0 license.

## Appendix

## Table of Contents:

Figure S1.
Figure S2.

bioRxiv preprint doi: https://doi.org/10.1101/2023.10.14.562349; this version posted October 1, 2024. The copyright holder for this preprint (which was not certified by peer review) is the author/funder. This article is a US Government work. It is not copyright under 17 USC 105 and is also made available for use under a CC0 license.

# Appendix Figure S1

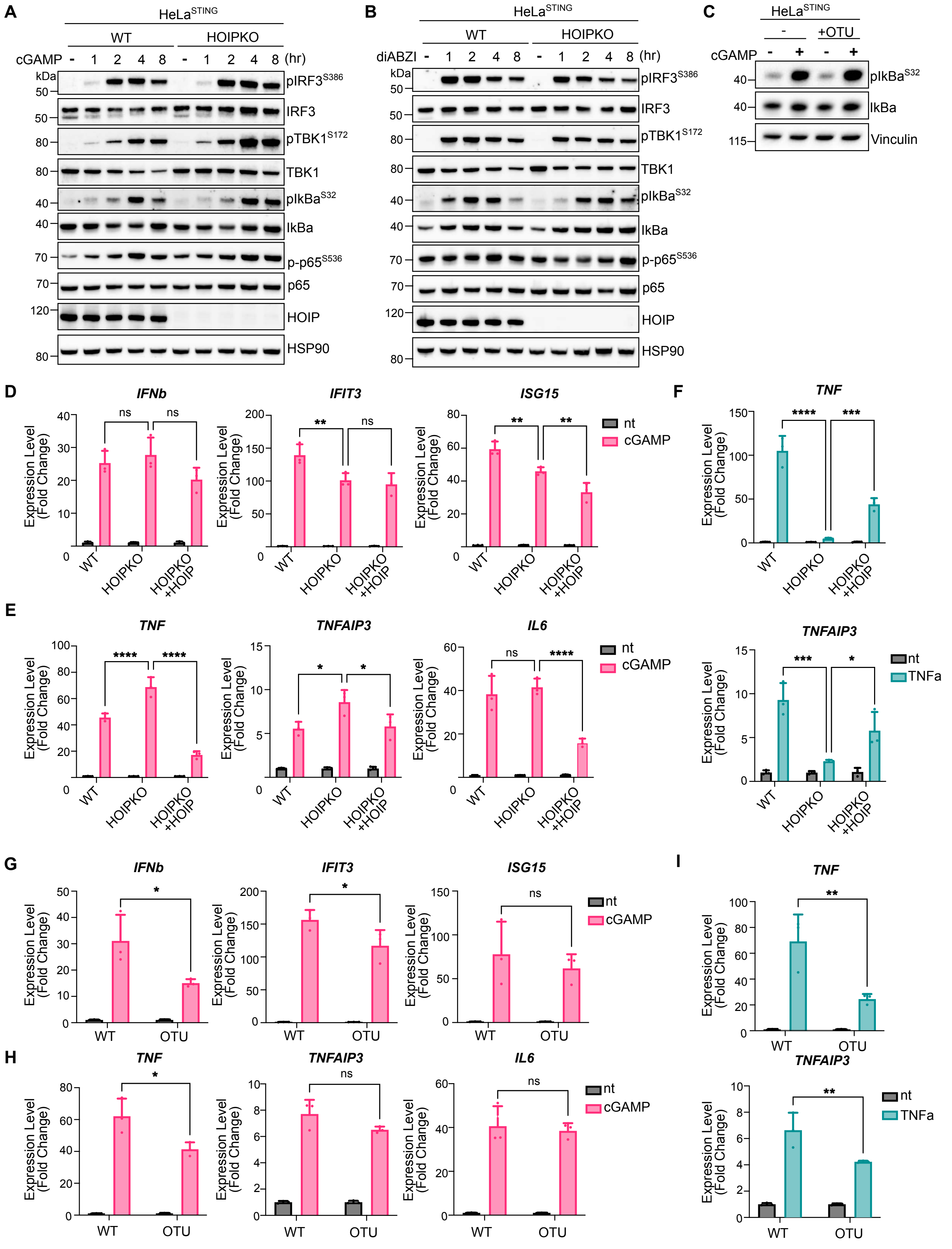

bioRxiv preprint doi: https://doi.org/10.1101/2023.10.14.562349; this version posted October 1, 2024. The copyright holder for this preprint (which was not certified by peer review) is the author/funder. This article is a US Government work. It is not subject to copyright under 17 USC 105 and is also made available for use under a CC0 license.

**Appendix Figure S1.**

**A-B**) Representative immunoblots of indicated proteins detected in WT and HOIPKO HeLa cell lysates prepared following treatment with 120 μg/mL cGAMP (A) or 1 μM diABZI (B) for 1, 2, 4, and 8 hours. Immunoblotting was replicated in 3 independent experiments.

**C**) Representative immunoblots of indicated proteins detected in lysates from $HeLa^{STING}$ and $HeLa^{STING}$ cells with stable overexpression of mEGFP-OTULIN prepared following treatment with 120 μg/mL cGAMP for 8 hours. Immunoblotting was replicated in 3 independent experiments.

**D-E**) Relative expression of indicated NFκB-related genes (D) and IRF3/interferon-related genes (E) detected by quantitative RT-PCR in $HeLa^{STING}$: WT, HOIPKO, and HOIPKO stably expressing mEGFP-HOIP cells treated with 120 μg/mL cGAMP for 8 hours. Quantification of relative expression is from 3 independent experiments analyzed at the same time. A 2-way ANOVA with a Tukey's multiple comparisons test was performed on $2^{-\Delta\Delta Ct}$ values. Error bars represent Standard Deviation. *<0.05, **<0.01, ***<0.001, ****<0.0001**F)** Relative expression of indicated NFκB-related genes detected by quantitative RT-PCR in $HeLa^{STING}$ WT,HOIPKO, and HOIPKO stably expressing mEGFP-HOIP cells treated with 10 ng/mL TNFα for 30 minutes. Quantification of relative expression is from 3 independent experiments analyzed at the same time. A 2-way ANOVA with a Tukey's multiple comparisons test was performed on $2^{-\Delta\Delta Ct}$ values. Error bars represent Standard Deviation. *<0.05, **<0.01, ***<0.001, ****<0.0001.

**F)** Relative expression of indicated NFκB-related genes detected by quantitative RT-PCR in $HeLa^{STING}$: WT, HOIPKO, and HOIPKO stably expressing mEGFP-HOIP cells treated with 10 ng/mL TNFα for 30 minutes. Quantification of relative expression is from 3 independent experiments analyzed at the same time. A 2-way ANOVA with a Tukey's multiple comparisons test was performed on $2^{-\Delta\Delta Ct}$ values. Error bars represent Standard Deviation. *<0.05, **<0.01, ***<0.001, ****<0.0001.

**G-H**) Relative expression of indicated NFκB-related genes (G) and interferon-related genes (H) detected by quantitative RT-PCR in WT $HeLa^{STING}$ and WT $HeLa^{STING}$ cells stably overexpressing mEGFP-OTULIN treated with 120 μg/mL cGAMP for 8 hours. Quantification of relative expression is from 3 independent experiments analyzed at the same time. A 2-way ANOVA with a Tukey's multiple comparisons test was performed on $2^{-\Delta\Delta Ct}$ values. Error bars represent Standard Deviation. *<0.05, **<0.01, ***<0.001, ****<0.0001.

bioRxiv preprint doi: https://doi.org/10.1101/2023.10.14.562349; this version posted October 1, 2024. The copyright holder for this preprint (which was not certified by peer review) is the author/funder. This article is a US Government work. It is not subject to copyright under 17 USC 105 and is also made available for use under a CC0 license.

**I)** Relative expression of indicated NFκB-related genes detected by quantitative RT-PCR in $HeLa^{STING}$ and $HeLa^{STING}$ cells with stable overexpression of mEGFP-OTULIN treated with 10 ng/mL TNFα for 30 minutes. Quantification of relative expression is from 3 independent experiments analyzed at the same time. A 2-way ANOVA with a Tukey's multiple comparisons test was performed on $2^{-\Delta\Delta Ct}$ values. Error bars represent Standard Deviation. *<0.05, **<0.01, ***<0.001, ****<0.0001.

bioRxiv preprint doi: https://doi.org/10.1101/2023.10.14.562349; this version posted October 1, 2024. The copyright holder for this preprint (which was not certified by peer review) is the author/funder. This article is a US Government work. It is not subject to copyright under 17 USC 105 and is also made available for use under a CC0 license.

# Appendix Figure S2

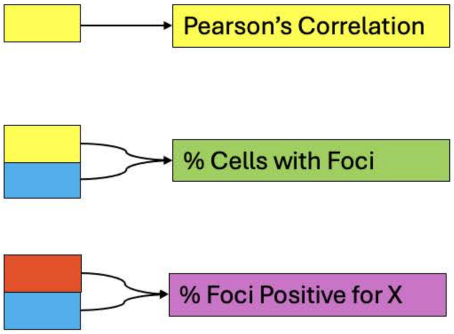


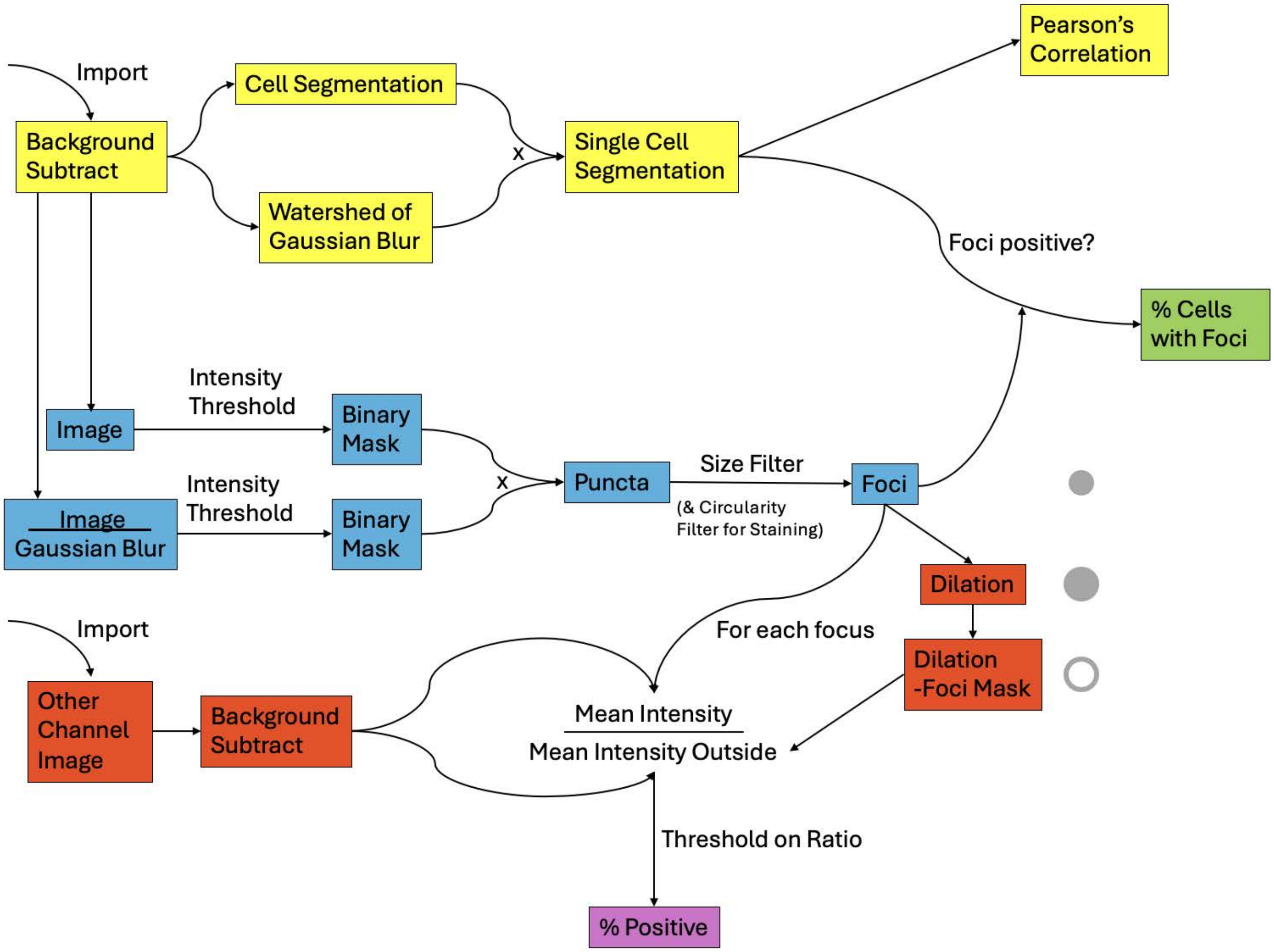

bioRxiv preprint doi: https://doi.org/10.1101/2023.10.14.562349; this version posted October 1, 2024. The copyright holder for this preprint (which was not certified by peer review) is the author/funder. This article is a US Government work. It is not subject to copyright under 17 USC 105 and is also made available for use under a CC0 license.

**Appendix Figure S2.**
Schematic of MATLAB workflow for image analysis.
