# Supplementary material for: STING induces LUBAC-mediated synthesis of linear ubiquitin chains to stimulate innate immune signaling
Source: bioRxiv. 2023 Oct 16:2023.10.14.562349. Preprint. [Version 2] doi: 10.1101/2023.10.14.562349 (PMC10592814; doi:10.1101/2023.10.14.562349)

bioRxiv preprint doi: https://doi.org/10.1101/2023.10.14.562349; this version posted October 16, 2023. The copyright holder for this preprint (which was not certified by peer review) is the author/funder. This article is a US Government work. It is not subject to copyright under 17 USC 105 and is also made available for use under a CC0 license.

Figure Legends

Supplementary Figure 1.

a) Representative spinning disk confocal images of $HeLa^{STING}$; mEGFP-LC3B cells treated with 120 µg/mL of cGAMP for 8 hours prior to PFA-fixation and immunostaining for mono- and poly-ubiquitin chains (Ub). Scale bar = 20 µm.

b) Quantification of the percentage (%) of cells positive for mEGFP-LC3B foci (left panel) and immunostained Ub foci (right panel) from experiments represented in Supp. Fig. 1a.

c) Quantification of the percentage (%) of mEGFP-LC3B foci with overlapping immunolabeled Ub+ signal from experiments represented in Supp. Fig. 1a. Quantification of imaging in b-c is from 3 wells analyzed in the same experiment. Imaging was replicated in 3 independent experiments.

bioRxiv preprint doi: https://doi.org/10.1101/2023.10.14.562349; this version posted October 16, 2023. The copyright holder for this preprint (which was not certified by peer review) is the author/funder. This article is a US Government work. It is not subject to copyright under 17 USC 105 and is also made available for use under a CC0 license.

d-e) Representative spinning disk confocal images of HeLa^{STING}; mEGFP-LC3B cells treated with 120 µg/mL of cGAMP for 8 hours prior to PFA-fixation and immunostaining for K48- and K63-ubiquitin chains. Scale bar = 20 µm.

f) Quantification of the percentage (%) of mEGFP-LC3B foci with overlapping immunolabeled K48- and K63-ubiquitin chain signal from experiments represented in Supp. Fig. 1d-e. Quantification of imaging in d-e is from 3 wells analyzed in the same experiment. Imaging was replicated in 3 independent experiments.

g) Pearson correlation coefficient of mScarletI-LC3B and Vx3-EGFP over time in the live imaging experiment represented in Movie 1. FRT/TREX HeLa cells stably expressing FRT/TO-DD-Vx3-EGFP, BFP-P2A-STING, and mScarletI-LC3B were incubated with 1ug/mL Doxycycline and 500nM Shield1 for 24h prior to treatment with 120 ug/mL cGAMP and imaging every 30 minutes for 12 hours on a spinning disk confocal microscope. Quantification is from 3 wells analyzed in the same experiment. Imaging was replicated in 2 independent experiments.

h) Representative immunoblot of the Human anti-Linear Ubiquitin chain antibody produced and validated by Genentech in Matsumoto et al., 2012 (*16*) from experiments also represented in Fig. 1b-e. Immunoblotting was replicated in 3 independent experiments.

Supplementary Figure 2.

a) Representative Airyscan-processed confocal images of HeLa^{STING}; mEGFP-LC3B (green) cells treated with 100uM Monensin for 1 hour prior to PFA-fixation and immunostaining with antibodies raised against mono- and poly-ubiquitin chains (Ub; magenta). Scale bar = 10 µm, and 2 µm (inset). Imaging was replicated in 2 independent experiments.

bioRxiv preprint doi: https://doi.org/10.1101/2023.10.14.562349; this version posted October 16, 2023. The copyright holder for this preprint (which was not certified by peer review) is the author/funder. This article is a US Government work. It is not subject to copyright under 17 USC 105 and is also made available for use under a CC0 license.

Supplementary Figure 3.

a-d) Representative immunoblots of indicated proteins detected in cell lysates from WT^{STING}, WT^{STING} stably expressing mEGFP-OTULIN, (a, c, and d), or WT^{STING}, $HOIPKO^{STING}$ (Clone #21), and $HOIPKO^{STING}$ (Clone #21) stably expressing HOIP (b) prepared following treatment with 120 μg/mL cGAMP for 8 hours. Immunoblotting was replicated in 3 independent experiments.

e) Representative immunoblots of endogenous STING detected in cell lysates from WT and HOIPKO (clone #21) HeLa cells without stable overexpression of STING. Cells were treated with 15 ug/mL cGAMP for 8 hours. Immunoblotting was replicated in 3 independent experiments.

bioRxiv preprint doi: https://doi.org/10.1101/2023.10.14.562349; this version posted October 16, 2023. The copyright holder for this preprint (which was not certified by peer review) is the author/funder. This article is a US Government work. It is not subject to copyright under 17 USC 105 and is also made available for use under a CC0 license.

Supplementary Figure 4.

a-b) Representative spinning disk confocal images of WT HeLa^{STING} cells stably expressing mScarletI-LC3B and either mEGFP-TNIP1- (**a**) or OPTN- (**b**) WT, LIR$_m$, -UBAN$_m$, or -Double$_m$ (LIR-UBAN$_m$) at the 10-hour time point represented in Figure 3c-d following treatment with 120 ug/mL cGAMP. Scale bar = 50 μm.

c) Representative spinning disk confocal images of WT and HOIPKO HeLa^{STING} cells stably expressing mScarI-LC3B (orange) cells treated with 120 μg/mL of cGAMP for 8 hours prior to PFA-fixation and immunostaining for TNIP1 (magenta) or OPTN (magenta). Scale bar = 50 μm.

d-e) Pearson correlation coefficient of mScarI-LC3B and endogenous TNIP1 (d) or OPTN (e) from experiments represented in Supp. Fig. 4c. Quantification is from 3 wells analyzed in the same experiment. Imaging was replicated in 2 independent experiments.

Supplementary Figure 5.

a) Relative expression of indicated NFκB-related genes detected by quantitative RT-PCR in WT^{STING}, HOIPKO^{STING} (Clone #21), and HOIPKO^{STING} (Clone #21) stably expressing mEGFP-HOIP cells treated with 10ng/mL TNFα for 30 minutes. Quantification of relative expression is from 3 independent experiments analyzed at the same time. A 2-way ANOVA with a Tukey's multiple comparisons test was performed on $2^{-\Delta\Delta Ct}$ values. Error bars represent Standard Deviation. *<0.05, **<0.01, ***<0.001, ****<0.0001.

bioRxiv preprint doi: https://doi.org/10.1101/2023.10.14.562349; this version posted October 16, 2023. The copyright holder for this preprint (which was not certified by peer review) is the author/funder. This article is a US Government work. It is not subject to copyright under 17 USC 105 and is also made available for use under a CC0 license.

b) Representative immunoblots of indicated proteins detected in lysates from WT^{STING}, HOIPKO^{STING} (Clone #21), and HOIPKO^{STING} (Clone #21) stably expressing untagged HOIP cells prepared following treatment with 120 ug/mL cGAMP for 8 hours. Immunoblotting was replicated in 3 independent experiments.

c-d) Relative expression of indicated NFκB-related genes (c) and interferon-related genes (d) detected by quantitative RT-PCR in WT^{STING}, HOIPKO^{STING} (Clone #21), and HOIPKO^{STING} (Clone #21) stably expressing mEGFP-HOIP cells treated with 120 ug/mL cGAMP for 8 hours. Quantification of relative expression is from 3 independent experiments analyzed at the same time. A 2-way ANOVA with a Tukey's multiple comparisons test was performed on $2^{-\Delta\Delta Ct}$ values. Error bars represent Standard Deviation. *<0.05, **<0.01, ***<0.001, ****<0.0001.

Supplementary Figure 6.

a) Relative expression of indicated NFκB-related genes detected by quantitative RT-PCR in HeLa^{STING} and HeLa^{STING} cells with stable overexpression of mEGFP-OTULIN treated with 10ng/mL TNFα for 30 minutes. Quantification of relative expression is from 3 independent experiments analyzed at the same time. A 2-way ANOVA with a Tukey's multiple comparisons test was performed on $2^{-\Delta\Delta Ct}$ values. Error bars represent Standard Deviation. *<0.05, **<0.01, ***<0.001, ****<0.0001.

b) Representative immunoblots of indicated proteins detected in lysates from HeLa^{STING} and HeLa^{STING} cells with stable overexpression of mEGFP-OTULIN prepared following treatment with 120 ug/mL cGAMP for 8 hours. Immunoblotting was replicated in 3 independent experiments.

c-d) Relative expression of indicated NFκB-related genes (c) and interferon-related genes (d) detected by quantitative RT-PCR in WT HeLa^{STING} and WT HeLa^{STING} cells stably overexpressing mEGFP-OTULIN treated with 120 ug/mL cGAMP for 8 hours. Quantification of relative expression is from 3 independent experiments analyzed at the same time. A 2-way ANOVA with a Tukey's multiple comparisons test was performed on $2^{-\Delta\Delta Ct}$ values. Error bars represent Standard Deviation. *<0.05, **<0.01, ***<0.001, ****<0.0001.

bioRxiv preprint doi: https://doi.org/10.1101/2023.10.14.562349; this version posted October 16, 2023. The copyright holder for this preprint (which was not certified by peer review) is the author/funder. This article is a US Government work. It is not subject to copyright under 17 USC 105 and is also made available for use under a CC0 license.

Supplementary Figure 7.

a) Representative Airyscan-processed confocal images of WT HeLa^{STING} cells stably expressing mEGFP-LC3B (green) treated with either DMSO, 10uM C53, 1uM diABZI, or both C53 and diABZI for 4 hours prior to PFA-fixation and immunostaining with antibodies raised against STING (magenta). Scale bar = 20 μm. Imaging was replicated in 2 independent experiments.

b-c) Representative spinning disk confocal images of FRT/TREX HeLa cells stably expressing FRT/TO-DD-Vx3-EGFP, BFP-P2A-STING, and mScarletI-LC3B at the 6-hour timepoint following treatment (b) and quantification of the percentage (%) of cells positive for Vx3-EGFP foci over time (c). Cells were incubated with 1ug/mL Doxycycline and 500nM Shield1 for 24h prior to treatment with either DMSO, 10uM C53, 1uM diABZI, or both C53 and diABZI, and imaging every 30 minutes for 12 hours on a spinning disk confocal microscope. Scale bar = 50 μm. Quantification is from 3 wells analyzed in the same experiment. Imaging was replicated in 2 independent experiments.

bioRxiv preprint doi: https://doi.org/10.1101/2023.10.14.562349; this version posted October 16, 2023. The copyright holder for this preprint (which was not certified by peer review) is the author/funder. This article is a US Government work. It is not subject to copyright under 17 USC 105 and is also made available for use under a CC0 license.

Supplementary Figure 8.

a-b) Representative spinning disk confocal images (a) and quantification of the percentage (%) of cells positive for immunostained TNIP1 foci (b) in WT HeLa^{STING} cells stably expressing mEGFP-LC3B (green) treated with either DMSO, 10uM C53, 1uM diABZI, or both C53 and diABZI for 4 hours prior to PFA-fixation and immunostaining for TNIP1 (magenta). Scale bar = 20 µm. Quantification is from 3 wells analyzed in the same experiment. Imaging was replicated in 2 independent experiments.

c-d) Representative spinning disk confocal images (c) and quantification of the percentage (%) of cells positive for immunostained OPTN foci (d) in WT HeLa^{STING} cells stably expressing mEGFP-LC3B (green) treated with either DMSO, 10uM C53, 1uM diABZI, or both C53 and diABZI for 4 hours prior to PFA-fixation and immunostaining for OPTN (magenta). Scale bar = 20 µm. Quantification is from 3 wells analyzed in the same experiment. Imaging was replicated in 2 independent experiments.

e-f) Representative immunoblots of indicated proteins detected in lysates from WT THP1 cells prepared following treatment with either DMSO, 10uM C53, 1uM diABZI, or both C53 and diABZI for 4 hours. Immunoblotting was replicated in 2-3 independent experiments.

Supplementary Figure 9.

a) Representative Airyscan-processed confocal images of HeLa^{STING} and HeLa^{STING} cells with stable overexpression of mEGFP-LC3B (green) treated with 120 µg/mL of cGAMP for 8 hours prior to PFA-fixation and immunostaining with antibodies raised against mono- and poly-ubiquitin chains (Ub; magenta) and STING (cyan). Scale bar = 20 µm, and 2 µm (inset). Imaging was replicated in 2 independent experiments.

b) Representative Airyscan-processed confocal images of WT HeLa^{STING} cells stably expressing mScarletI-LC3B alone or with stable expression of mEGFP-SopF (green) treated with 120 µg/mL of cGAMP for 8 hours prior to PFA-fixation and immunostaining with antibodies raised against mono- and poly-ubiquitin chains (Ub; magenta). Scale bar = 20 µm, and 2 µm (inset). Imaging was replicated in 3 independent experiments.

bioRxiv preprint doi: https://doi.org/10.1101/2023.10.14.562349; this version posted October 16, 2023. The copyright holder for this preprint (which was not certified by peer review) is the author/funder. This article is a US Government work. It is not subject to copyright under 17 USC 105 and is also made available for use under a CC0 license.

Movie 1.

Representative spinning disk confocal movie of FRT/TREX HeLa cells stably expressing FRT/TO-DD-Vx3-EGFP, BFP-P2A-STING, and mScarletI-LC3B. Cells were incubated with 1ug/mL Doxycycline and 500nM Shield1 for 24h prior to treatment with 120 ug/mL cGAMP and imaging every 30 minutes for 12 hours on a spinning disk confocal microscope. Scale bar = 25 μm.

bioRxiv preprint doi: https://doi.org/10.1101/2023.10.14.562349; this version posted October 16, 2023. The copyright holder for this preprint (which was not certified by peer review) is the author/funder. This article is a US Government work. It is not subject to copyright under 17 USC 105 and is also made available for use under a CC0 license.

Supplementary Figure 1.

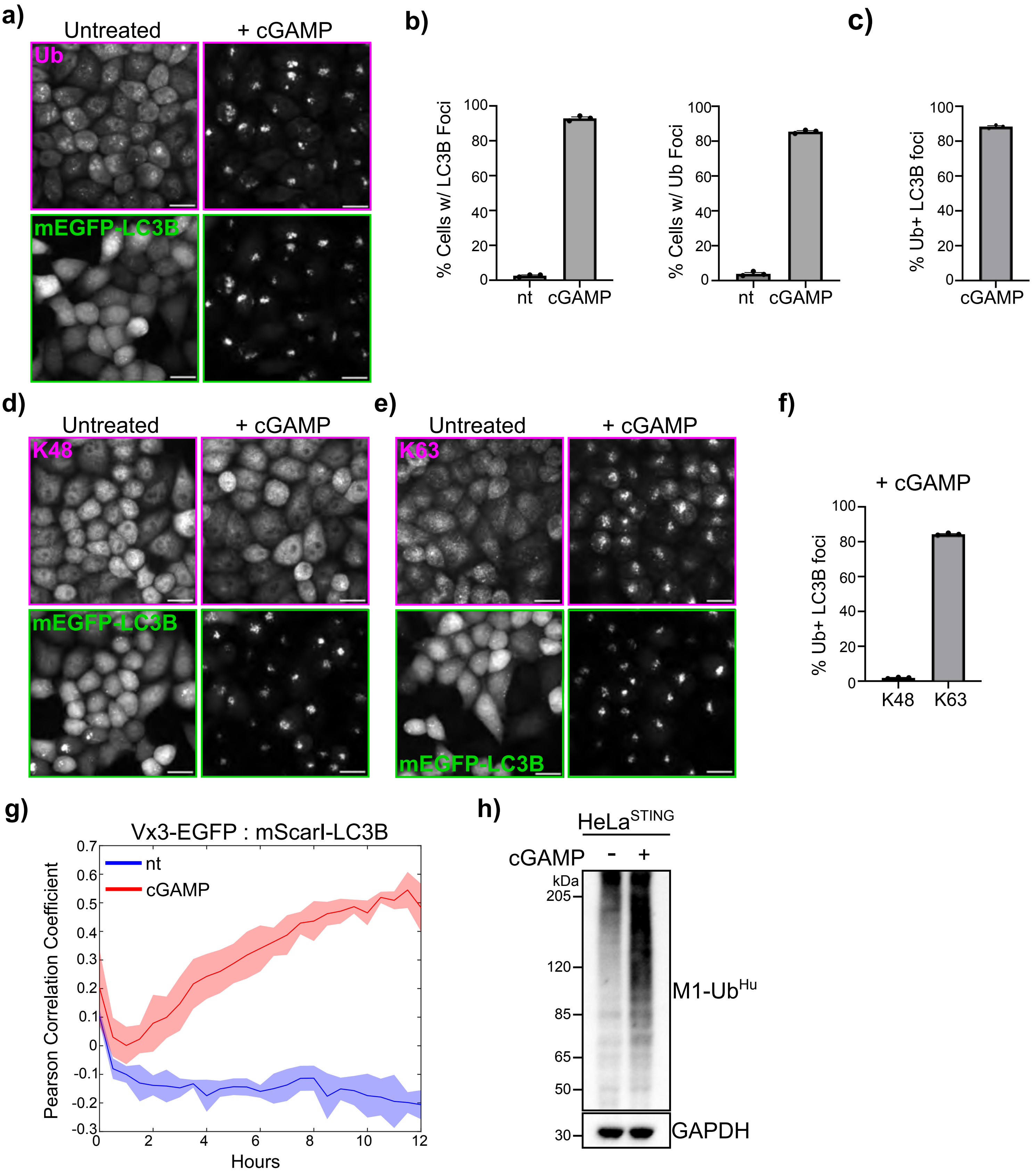

Supplementary Figure 2.

bioRxiv preprint doi: https://doi.org/10.1101/2023.10.14.562349; this version posted October 16, 2023. The copyright holder for this preprint (which was not certified by peer review) is the author/funder. This article is a US Government work. It is not subject to copyright under 17 USC 105 and is also made available for use under a CC0 license.

a)

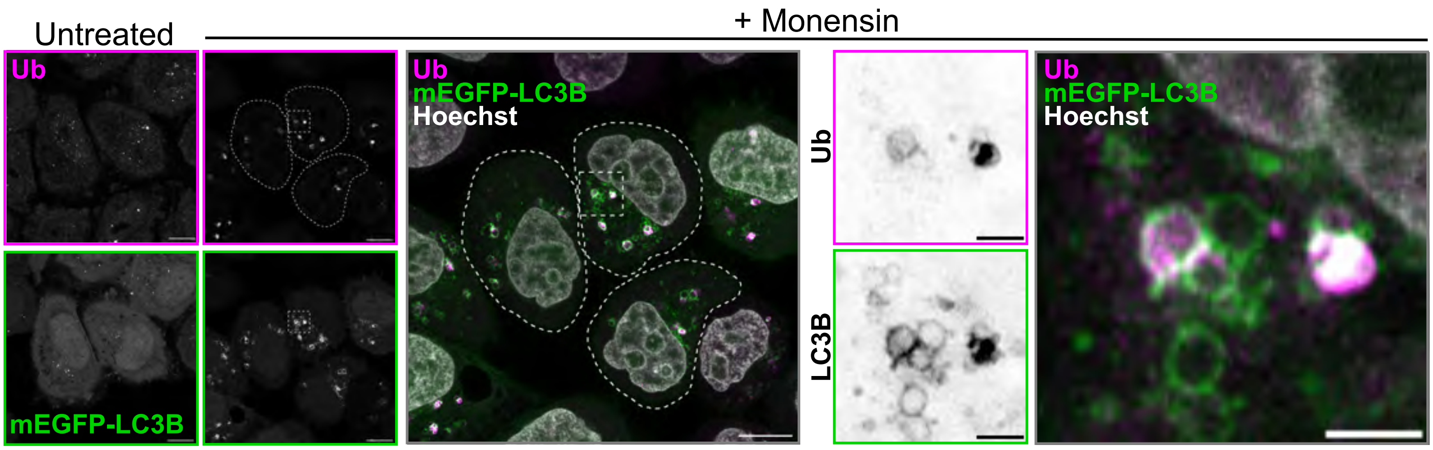

bioRxiv preprint doi: https://doi.org/10.1101/2023.10.14.562349; this version posted October 16, 2023. The copyright holder for this preprint (which was not certified by peer review) is the author/funder. This article is a US Government work. It is not subject to copyright under 17 USC 105 and is also made available for use under a CC0 license.

Supplementary Figure 3.

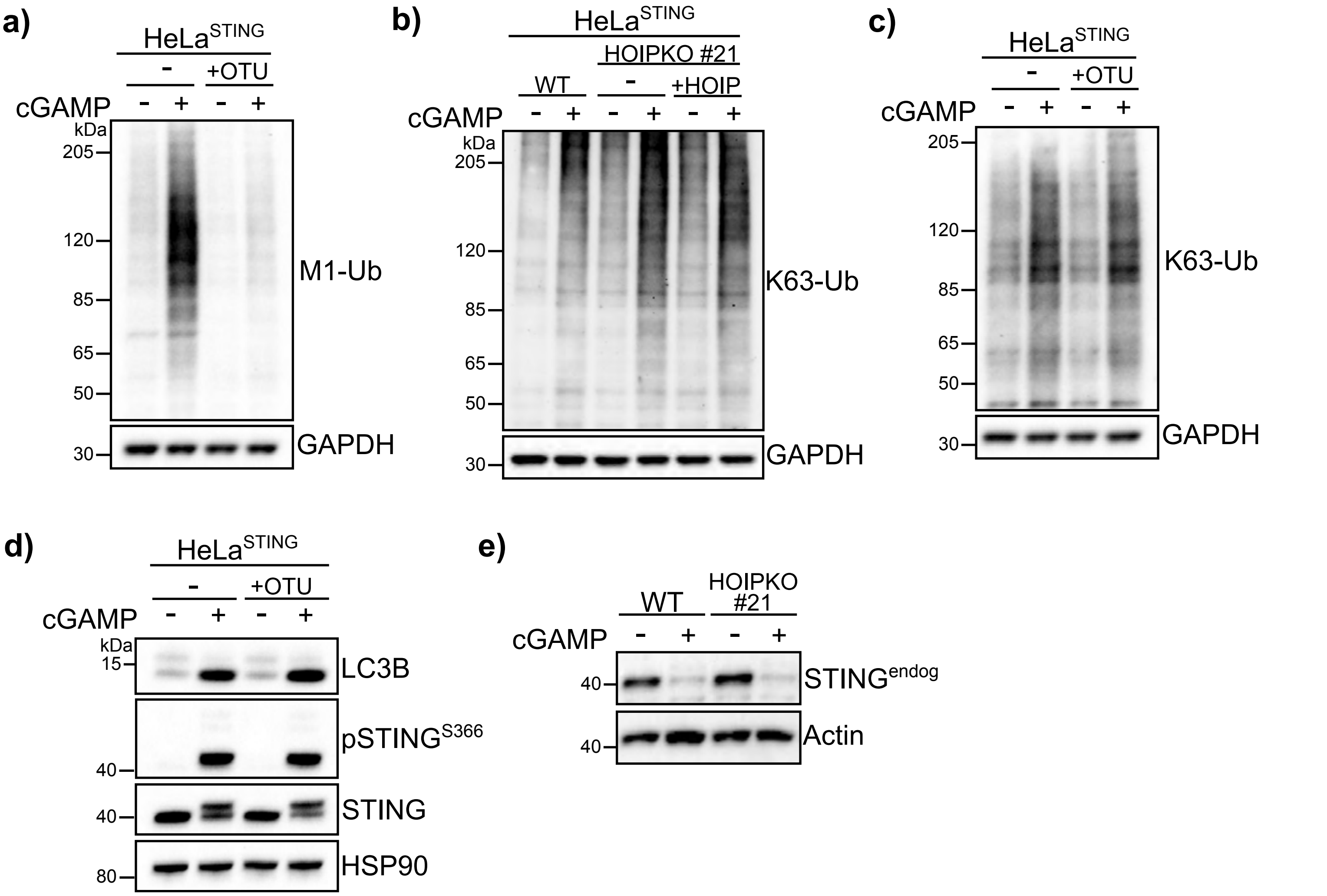

bioRxiv preprint doi: https://doi.org/10.1101/2023.10.14.562349; this version posted October 16, 2023. The copyright holder for this preprint (which was not certified by peer review) is the author/funder. This article is a US Government work. It is not subject to copyright under 17 USC 105 and is also made available for use under a CC0 license.

Supplementary Figure 4.

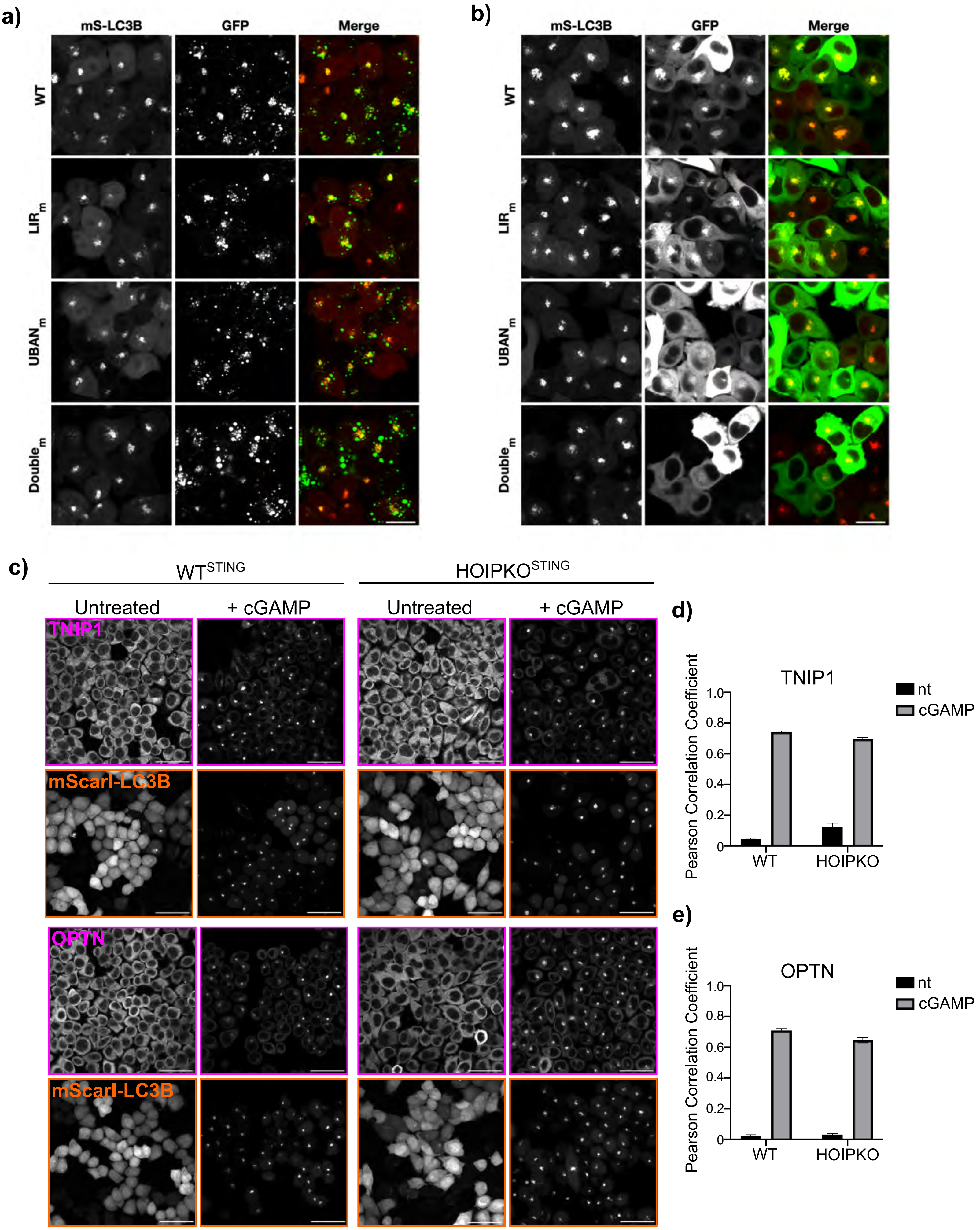

bioRxiv preprint doi: https://doi.org/10.1101/2023.10.14.562349; this version posted October 16, 2023. The copyright holder for this preprint (which was not certified by peer review) is the author/funder. This article is a US Government work. It is not subject to copyright under 17 USC 105 and is also made available for use under a CC0 license.

Supplementary Figure 5.

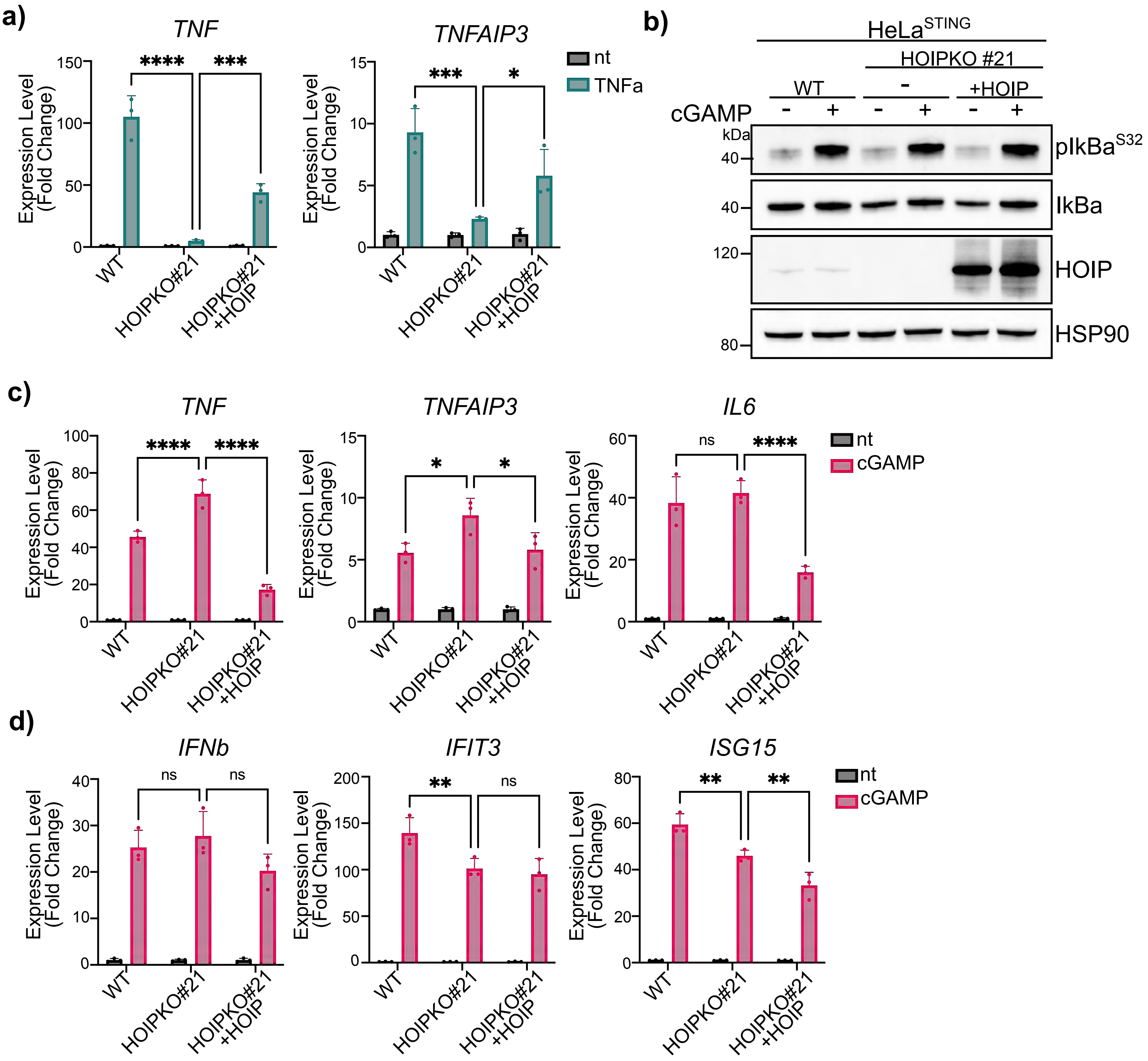

bioRxiv preprint doi: https://doi.org/10.1101/2023.10.14.562349; this version posted October 16, 2023. The copyright holder for this preprint (which was not certified by peer review) is the author/funder. This article is a US Government work. It is not subject to copyright under 17 USC 105 and is also made available for use under a CC0 license.

Supplementary Figure 6.

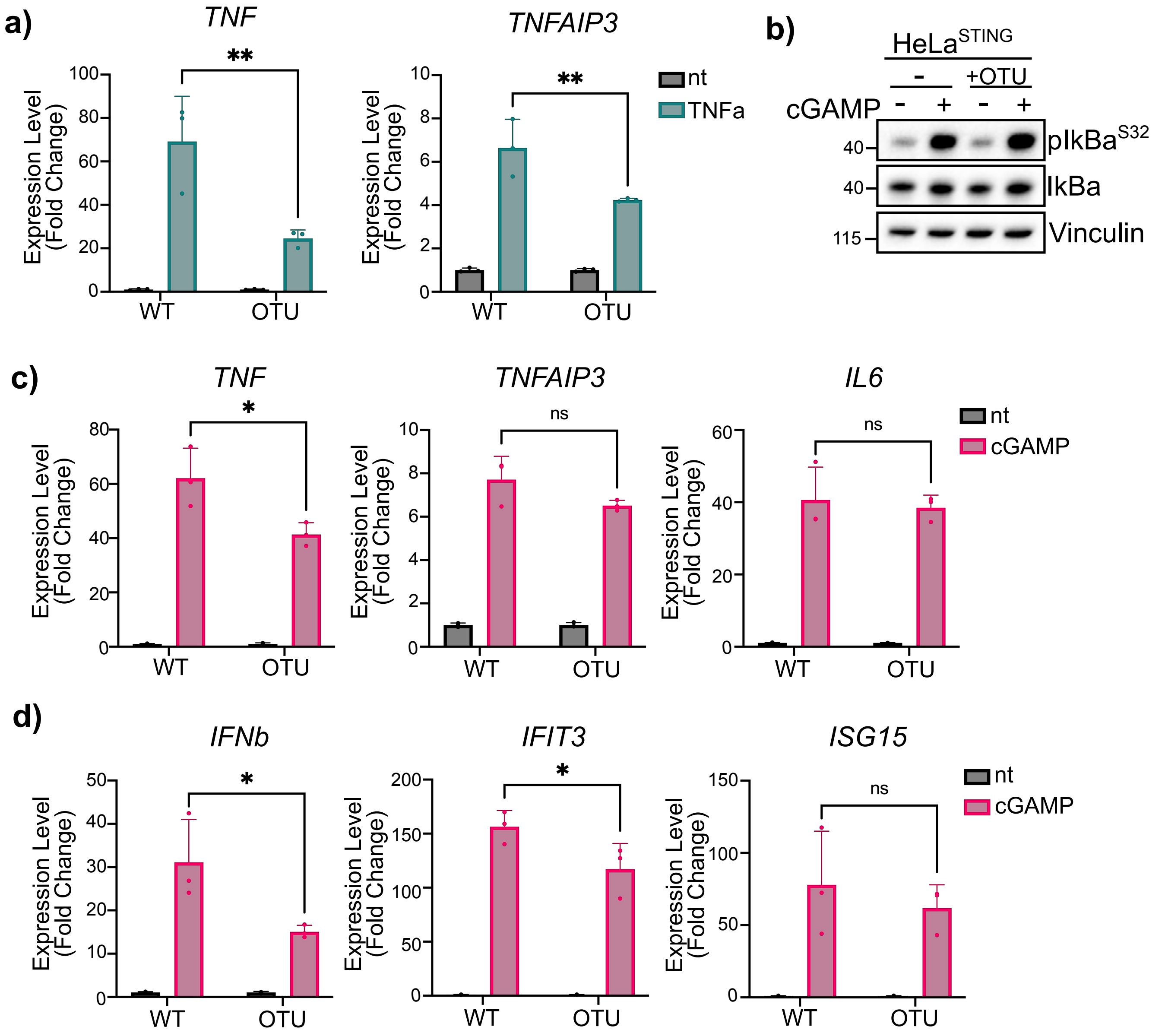

bioRxiv preprint doi: https://doi.org/10.1101/2023.10.14.562349; this version posted October 16, 2023. The copyright holder for this preprint (which was not certified by peer review) is the author/funder. This article is a US Government work. It is not subject to copyright under 17 USC 105 and is also made available for use under a CC0 license.

Supplementary Figure 7.

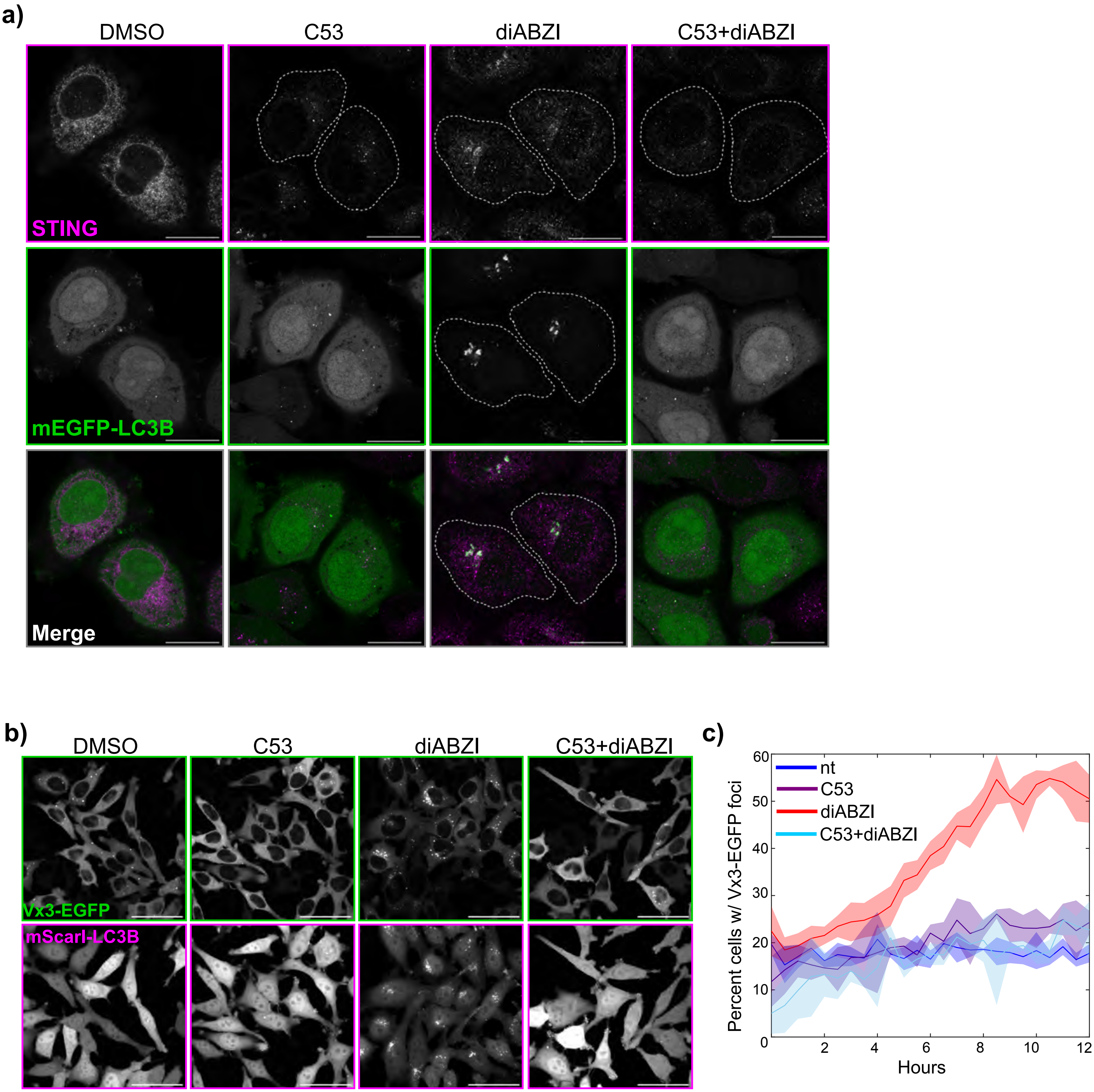

bioRxiv preprint doi: https://doi.org/10.1101/2023.10.14.562349; this version posted October 16, 2023. The copyright holder for this preprint (which was not certified by peer review) is the author/funder. This article is a US Government work. It is not subject to copyright under 17 USC 105 and is also made available for use under a CC0 license.

Supplementary Figure 8.

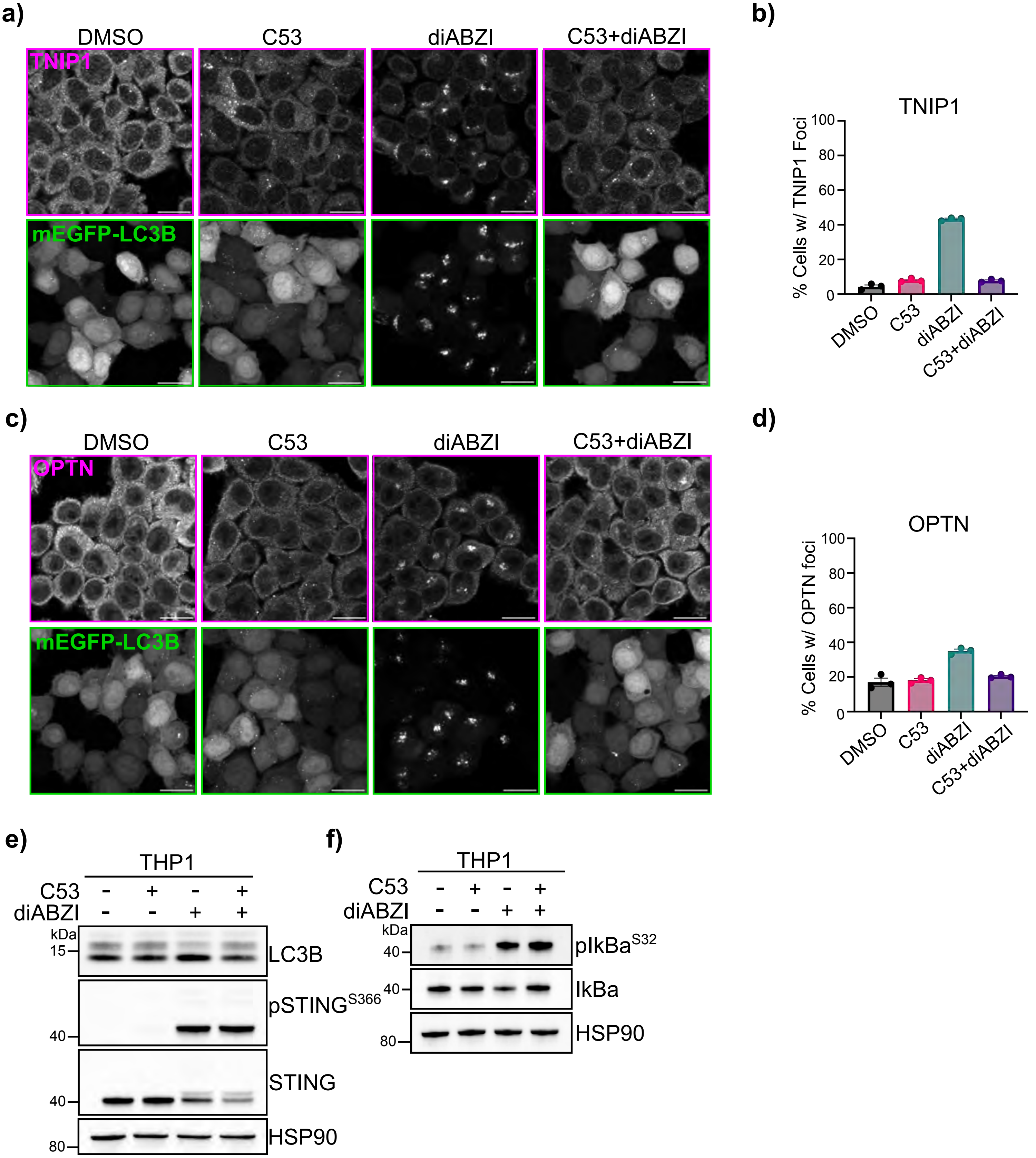

bioRxiv preprint doi: https://doi.org/10.1101/2023.10.14.562349; this version posted October 16, 2023. The copyright holder for this preprint (which was not certified by peer review) is the author/funder. This article is a US Government work. It is not subject to copyright under 17 USC 105 and is also made available for use under a CC0 license.

Supplementary Figure 9.

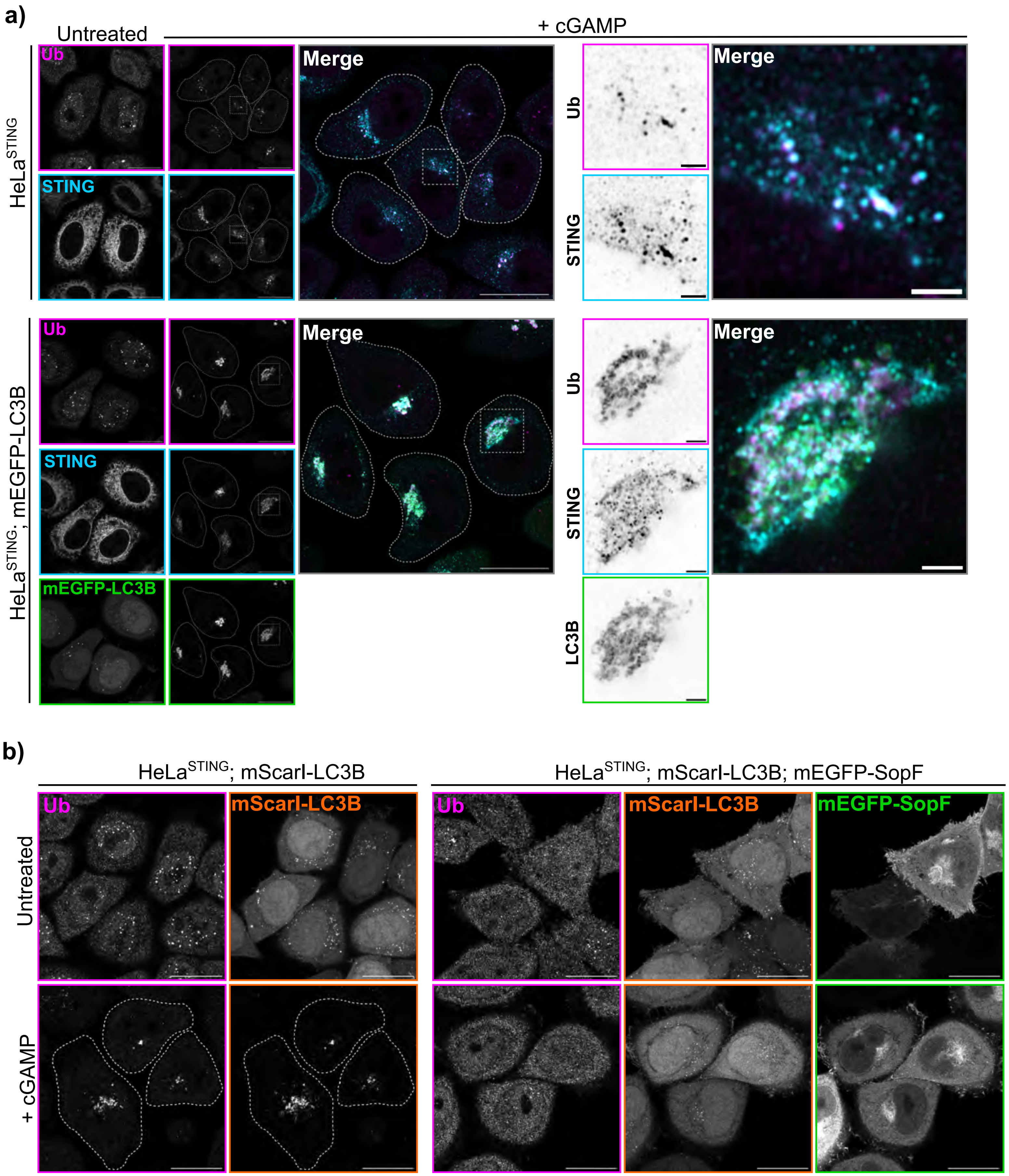

Supplement: Supplementary file 1 [file NIHPP2023.10.14.562349v2-supplement-3.pdf]
